# Interplay between Structure and Conduction Mechanism of Piperazinium‐Functionalized Poly[Ethylene Pyrrole/Ethylene Ketone/Propylene Ketone] Anion Conducting Membranes

**DOI:** 10.1002/cssc.202402765

**Published:** 2025-05-05

**Authors:** Afaaf Rahat Alvi, Keti Vezzù, Francesco Lanero, Paolo Sgarbossa, Angeloclaudio Nale, Vito Di Noto

**Affiliations:** ^1^ Department of Industrial Engineering Section of Chemistry for Technology University of Padova Via F. Marzolo 9 I‐35131 Padova Italy; ^2^ UDR di Padova INSTM Via F. Marzolo 9 I‐35131 Padova Italy

**Keywords:** anion membranes, conductivities, functionalized polyketones, high alkaline stabilities, X‐ray diffraction studies

## Abstract

Anion exchange membranes (AEMs) have been proposed as potential alternatives to proton exchange membranes in electrochemical energy conversion and storage devices. Herein, the synthesis of a series of AEMs based on random piperazinium‐methyl N‐substituted poly[ethylene pyrrole/ethylene ketone/propylene ketone] (P‐FPKK) and their structural characterization together with the elucidation of the ion conduction mechanism are reported. Different copolymers are prepared by a Paal–Knorr reaction of a poly(ethylene ketone/propylene ketone) copolymer precursor with 1‐(2‐aminoethyl) piperazine, modulating the molar ratios amine/(1,4‐diketonic repeat units of copolymer) from ¼ to 3. The composition and structure are investigated by elemental analysis, attenuated total reflectance–Fourier transform infrared spectroscopy, nuclear magnetic resonance, and X‐ray diffraction. The degree of functionalization, *f*, plays a crucial role in controlling the thermal, morphological, and electrochemical properties of the P‐FPKK_f_(I)_g_ and P‐FPKK_f_(OH)_g_ membranes. P‐FPKK_0.49_(OH)_0.45_ displays an ionic conductivity of 11.3 mS cm^−1^ at 60 °C. After alkaline aging (1 m KOH at 60 °C for 336 h), retaining its structural integrity and 25% of its initial ionic conductivity value, suggesting them as possible candidates for testing in alkaline energy conversion and storage devices. Furthermore, broadband electrical spectroscopy studies reveal that the long‐range charge migration processes are modulated by the segmental motion of the backbone chains of the copolymer matrix.

## Graphical abstract

A new anion exchange membrane based on random piperaziniumethyl N‐substituted poly[ethylene pyrrole/ethylene ketone/propylene ketone] (P‐FPKK), with a conductivity of 11.3 mS cm^−1^ at 60 °C in hydroxyl form, is developed. This material is very promising to overcome the issue of anionic membranes as potential alternatives to proton exchange membranes in electrochemical energy conversion and storage devices.

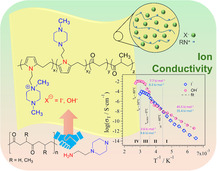

## 1 Introduction

The major challenge for the energy sector in recent years is the development of sustainable renewable wind and solar energy sources.^[^ ^1^ ^]^ However, their wide practical implementation is limited by their intermittent nature and often by the restrictions imposed by the geographical installation site. Energy storage systems are crucial to mitigating these latter drawbacks and to reach steady energy outputs.^[^ ^2^, ^3^ ^]^ Thus, extensive research is carried out to develop cost‐effective, stable, environmentally friendly, and reliable electrochemical energy conversion and storage devices such as fuel cells, batteries, and supercapacitors based on ion‐conducting polymers.^[^ ^4^, ^5^, ^6^, ^7^ ^]^ Proton exchange membranes (PEMs) and anion exchange membranes (AEMs), which act as both separators and as ion‐conductive materials between the anode and the cathode of a cell, are key components for the above devices.^[^ ^8^, ^9^, ^10^, ^11^, ^12^ ^]^ It is reported by Varcoe et al.^[^ ^13^ ^]^ that the performance and the cost of the energy storage and conversion devices are largely dependent on the membranes. For these reasons, in the past two decades, most of the effort has been focused on the development of a new PEM alternative to the perfluorinated ionomers, such as Nafion, and others. These latter, despite their high cost, show excellent chemical, thermal, and mechanical stability and ionic conductivity.^[^ ^14^, ^15^, ^16^ ^]^

It was proposed that AEMs are promising alternatives to PEMs for applications in low‐temperature fuel cells owing to their reduced fuel crossover, minimized water management at the electrodes, low cost, and enhanced kinetics in the oxygen reduction reaction by means of nonprecious metal electrocatalysts. In addition, the replacement of traditional alkaline liquid electrolytes (i.e., KOH solutions) with solid‐state AEMs mitigates the effect of carbonate precipitation, which compromises the conductivity of the membranes and blocks the pores of the gas diffusion layer inhibiting the overall performance of the fuel cell.^[^ ^17^ ^]^

AEMs^[^ ^18^ ^]^ suitable for practical applications require: 1) a high hydroxide conductivity; 2) a simple and versatile synthesis of ionomers and membranes; 3) low cost; and 4) a good chemical, thermal, mechanical, and electrochemical stability.^[^ ^17^ ^]^ Unfortunately, with respect to PEMs, most of proposed AEMs suffer from low ionic conductivity due to the inherently lower mobility of the hydroxide anion, 20.64 × 10^−8^ m^2^ s^−1 ^V^−1^ versus 36.23 × 10^−8^ m^2^ s^−1 ^V^−1^ of protons, and a low chemical and electrochemical stability in alkaline conditions.^[^ ^13^ ^]^ Several strategies have been explored to increase the stability and anion conductivity of AEMs by modulating the chemistry of polar side groups, polymer backbone chains,^[^ ^19^, ^20^, ^21^ ^]^ crosslinking processes,^[^ ^20^, ^22^ ^]^ and mesoscale nanophase morphologies.^[^ ^13^, ^23^ ^]^

Typically, AEMs are based on polar side chains containing ammonium (–NR_3_^+^),^[^ ^24^ ^]^ phosphonium (–PR_3_^+^),^[^ ^25^ ^]^ and sulfonium (–SR_2_^+^) groups.^[^ ^26^ ^]^ To date, the AEMs based on quaternary ammonium side chains are the most intensively studied.^[^ ^27^, ^28^ ^]^ The strategy to introduce quaternary ammonium side groups into the membrane matrix consists of the immersion of the preformed membranes endowed: 1) with tertiary amine side groups into a haloalkyl solution^[^ ^8^, ^29^ ^]^ or 2) with haloalkyl side chains into a trimethylamine aqueous solution.^[^ ^30^, ^31^ ^]^

The aim to obtain new AEMs with high ionic conductivity, acceptable water uptake, reduced swelling, good mechanical stability, and low‐cost prompts research activities to continuously improve and explore innovative synthesis routes. Among these, the Paal–Knorr reaction of 1,4‐dicarbonyl repeat units of backbone aliphatic polyalkylketones chains with a selected primary amine seems to provide promising membranes with excellent thermal and electrochemical properties, as demonstrated in the pioneering works by the group of Di Noto et al.^[^ ^8^, ^32^, ^33^, ^34^, ^35^ ^]^ This method allowed to devise membranes with suitable N‐functional side groups, very effective in also the modulation of physicochemical and electrochemical properties^[^ ^8^, ^34^ ^]^ of AEMs.

Relevant advantages of this simple and flexible synthesis protocol are: 1) the absence of any type of catalyst and 2) the soft preparation conditions, which allow the formation of ionomers with ethylene pyrrolic repeat units functionalized with the desired polar pendant groups.^[^ ^36^, ^37^ ^]^

**Scheme 1:**
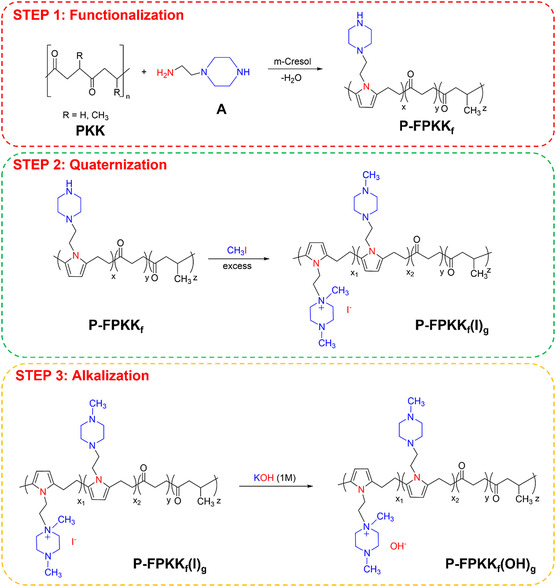
Synthesis of poly{[(1,4‐dimethyl piperaziniumethyl)‐N‐ethylene pyrrole(X)/(4‐methyl piperazineethyl)]‐N‐ethylene pyrrole/(ethylene ketone)/(propylene ketone)}, P‐FPKK_f_(X)_g_, where $f=\frac{x}{x+y+z}$, $x=x_{1}+x_{2}$, and $g=\frac{x_{1}}{x+y+z}$. Variables *x*_1_, *x*_2_, *y*, and *z* are the moles of [(1,4‐dimethyl piperaziniumethyl)]‐N‐ethylene pyrrole, [(4‐methyl piperazineethyl)]‐N‐ethylene pyrrole, ethylene ketone, and propylene ketone, respectively.

On this basis, here, the aim is: 1) to obtain new AEMs by functionalizing random polyalkylketones with very stable polar side chains and 2) to investigate the correlations existing in the prepared materials between composition, physicochemical properties, and conductivity. In the first step, functionalized polyketone (FPKK) precursors are prepared by reacting a poly(ethylene ketone/propylene ketone) (PKK) copolymer (“the terpolymer”) with 1‐(2‐aminoethyl) piperazine (A). The target is to obtain an FPKK precursor bearing ethyl piperazine pendant side chains attached to the N pyrrolic rings of the backbone chains (see **Scheme** **1**, step 1). In the second step, by reaction of P‐FPKK_f_ with methyl iodide (CH_3_I), the piperazine side groups are quaternized into stable piperazinium cations, whose iodide counteranions can be exchanged to hydroxide ones by immersion in a KOH aqueous solution (step 3).

The degree of functionalization of FPKK precursors is modulated by varying the A/PKK molar ratio in the reagent mixture. The structural features of precursors and ionomers were studied by nuclear magnetic resonance spectroscopy (NMR) (^1^H, ^13^C, and ^1^H‐^13^C HSQC (heteronuclear single‐quantum correlation)), attenuated total reflectance–Fourier‐transform infrared spectroscopy (ATR–FTIR), and by wide‐angle X‐ray diffraction (WAXD) studies. The stoichiometry and conversion of ethylene carbonyl repeat units into pyrrole ethylene units in samples was determined by CHNS/O elemental analyses. The thermal stability and transitions were studied by thermogravimetric analysis (TGA) and modulated differential scanning calorimetry (MDSC), respectively. Broadband electrical spectroscopy (BES) studies are conducted to investigate the electrical response and to clarify the conductivity mechanisms of the materials.

## 2 Results and Discussion

### 2.1 Compositional Analysis

The preparation of the membranes in I^−^ and OH^−^ form (**Scheme** **1**) was conducted in three steps. Step 1 describes the synthesis of P‐FPKK_f_. The degree of conversion of 1,4‐diketonic units into ethylene pyrrole repeat units (*f* degree of functionalization) is determined by elemental analyses using:^[^ ^8^ ^]^

(1) $$f=\frac{g_{N}\cdot {\text{MW}}_{\text{PKK}}}{n\cdot {\mathrm{MW}}_{N}-g_{N}({\text{MW}}_{\text{FP}}-{\text{MW}}_{\text{PKK}})}$$

where *g*_*N*_ and MW_*N*_ (14.0067 g mol^−1^) are the nitrogen fraction and atomic weight, respectively. *n* is the number of N atoms in a repeat unit. MW_PKK_ is the molecular weight of PKK repeat units (113.79 g mol^−1^). MW_FP_ is the molecular weight of an N‐substituted ethylene pyrrole repeat unit of Scheme 1 (Step 1).

**Table** **1** summarizes the stoichiometry on the degree of functionalization, *f*, of P‐FPKK_f_ and P‐FPKK_0.49_(I)_0.45_ membranes (Scheme 1). Results reveal that *f* (see Equation 2) increases with the (moles of amine derivative, A)/(1,4‐diketonic repeat units) molar ratio.

(2) $$f=\frac{x}{x+y+z}$$

**Table 1:** Stoichiometry of P‐FPKK_f_ at various degrees of functionalization, *f*, and of P‐FPKK_0.49_(I)_0.45_. Values are determined by CHNS/O elemental analyses. *x* are the moles of functionalized ethylene pyrrole repeat units. *y* and *z* are the moles of ethylene ketone and propylene ketone repeat units, respectively. *g* is the molar fraction of repeat units with quaternized polar groups on the total amount of repeat units of polymer and *Q*% is the percentage of quaternized side chains on the ethylene pyrrole‐functionalized groups (see caption of Scheme 1).

Sample	A/PK	%N	%C	%H	*x*	*y*+*z*	*f*	*g*	*Q*%
P‐FPKK_0.19_	0.25/1	5.79a)	68.4a)	7.48a)	0.19a)	0.80a)	0.19b)	/c)	/d)
P‐FPKK_0.28_	0.5/1	7.98	69.37	8.00	0.28	0.72	0.28	/	/
P‐FPKK_0.49_	1/1	12.5	71.1	8.30	0.49	0.51	0.49	/	/
P‐FPKK_0.70_	3/1	16.14	72.21	8.69	0.70	0.30	0.70	/	/
P‐FPKK_0.19_(I)_0.18_	0.25/1	5.00	58.70	7.60	0.19	0.81	0.19	0.18	95.2%
P‐FPKK_0.28_(I)_0.27_	0.5/1	6.40	56.50	7.45	0.28	0.72	0.28	0.27	96.0%
P‐FPKK_0.49_(I)_0.45_	1/1	8.58	52.62	6.03	0.49	0.61	0.49	0.45	91.8%
P‐FPKK_0.70_(I)_0.66_	3/1	10.6	50.00	7.03	0.70	0.30	0.71	0.67	94.1%

a) Determined by CHNS/O analyses;

b) $f=\frac{x}{x+y+z}$, the degree of functionalization;

c) $g=\frac{x_{1}}{x+y+z}$;

d) $Q\%=\frac{x_{1}}{x_{1}+x_{2}}\cdot 100$.

**Figure 1:**
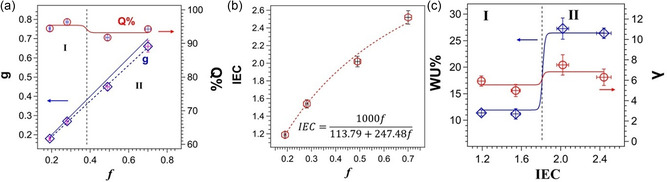
a) Dependence of *g* and *Q*% on *f*. *g* is the molar fraction of repeating units with quaternized polar groups on the total amount of repeating units of polymers. *Q*% is the percentage of quaternized repeating units to the total of functionalized ethylene pyrrole repeat units. b) IEC versus *f* of PKK_f_(*X*)_g_ membranes. The dotted line corresponds to the profile determined by Equation (3), assuming MW_PKK_ = 113.79 and MW_QP_ = 361.27 g mol^−1^. c) Dependence of WU% and *λ* on IEC. Regions I and II delimited by IEC = 1.8 and *f* = 0.39 are reported. The error bars of the experimental data are reported in all the graphs.

The dependence as a function of *f* of *g* and the *Q*% of membranes in iodide form is shown in **Figure** **1**a. *g* is the fraction of quaternized polar groups (1,4‐dimethyl piperaziniumethyl groups, see Section S1, Supporting Information) formed in Step 2 to the total repeating units of the polymer and *Q*% is the percentage of quaternized polar side chains to the total functionalized repeating units.

The dependence on *f* of the ion exchange capacity (IEC), determined by elemental analyses (Table S1, Supporting Information), is shown in Figure 1b. Particularly, IEC of the membranes in iodide form increases with *f* according to Equation (3). The IEC versus *f* relationship can be determined as follows, with MW_PKK_ the molecular weight of the unmodified PKK diketonic repeat unit (113.79 g mol^−1^) and MW_QP_ the quaternized ethylene pyrrole repeat unit in iodide form (361.27 g mol^−1^).

Average MW of the polymer repeat units: $\bar{\text{MW}}$_RU_ = MW_PKK_⋅(1 − *f*) + MW_QP_ ⋅ *f*

Average number of moles of polymer per gram: *n*_pol_ = $\frac{1}{{\bar{\text{MW}}}_{\text{RU}}}=\frac{1}{{\text{MW}}_{\text{PKK}}\cdot \left(1-f\right)\;+{\text{ MW}}_{\text{QP}}\cdot f}$

Number of moles (equivalents) of quaternized units: *n*_*i*on_ = $\frac{1}{{\text{MW}}_{\text{PKK}}\cdot \left(1-f\right)+{\text{MW}}_{\text{QP}}\cdot f}\cdot f$

(3) $$\text{IEC}(\text{meq/g})=\frac{n_{\text{ion}}\cdot 1000}{1}=\frac{f\cdot 1000}{MW_{\text{PKK}}\cdot (1-f)+MW_{\text{QP}}\cdot f}=\frac{f\cdot 1000}{MW_{\text{PKK}}+f\cdot (MW_{\text{QP}}-MW_{\text{PKK}})}$$

For simplicity and based on high degree of quaternization of the membranes (always higher than 90%, see Table 1) *g* = *f* has been considered as an approximation.

The profiles of water uptake (WU%) and hydration number (*λ*) versus IEC of membranes at room temperature are shown in Figure 1c (Table S1, Supporting Information). In electrochemical energy conversion devices such as fuel cells, the hydration parameters are crucial for the selection of the AEMs suitable for practical applications. Indeed, these parameters summarize the delicate balance existent between the morphology, structure, and composition of membranes and their water absorption thermodynamics.

A high value of water uptake results in swelling of the AEMs, thus compromising the mechanical stability and performance of materials.^[^ ^38^ ^]^ The dependence of water uptake and *λ* on the IEC presents a sigmoidal profile, suggesting that two compositional and morphological regions (I and II) are present in these materials, which are delimited by the value of IEC ≈ 1.8 (see Figure 1c). At IEC < 1.8, the average values observed for WU% and *λ* are ≈12% and 5, respectively, while at IEC > 1.8 the values are 27% and 7, respectively.

This demonstrates that in region I and II, the mesoscale microstructure of the membranes is correlated to two different phenomena: 1) the average conformational geometry of backbone chains and 2) the interchain interactions between side polar chains. Regarding IEC, the ionic conductivity at room temperature (RT) shows behaviors concurring with that of WU% and *λ* (see Table S1, Supporting Information).

Results reveal that (Figure 1): 1) in region I, *g* ≈ *f*, while in II, *g* < *f*; and 2) *Q*% is generally higher than 90% and shows a sigmoidal behavior on *f* with a step at approximately *f* = 0.39. This indicates that in region I side groups are almost all quaternized, while in region II an average of ≈6% of side chains are not quaternized, suggesting a combined effect of the different microstructure of the polymer and the inhibiting effect of the proximity repulsion between the cationic groups along the same polymer chain at *f* > 0.39.

Taken all together, these results suggest that both the electric response and physicochemical properties of membranes are expected to be significantly different in region II with respect to I region.

### 2.2 Thermal Stability Study

**Figure 2:**
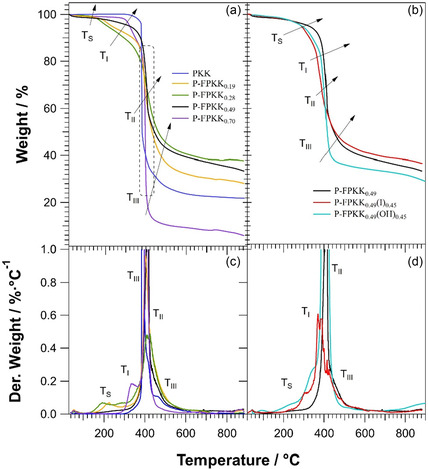
a) Selected HR‐TGA profiles of pristine polyketone (PKK) and P‐FPKK_f_ at different degrees of functionalization, *f.* b) HR‐TGA profiles of P‐FPKK_0.49_, P‐FPKK_0.49_(I)_0.45_, and P‐FPKK_0.49_(OH)_0.45_ membranes in the dry state. c) and d) The first derivative of the HR‐TGA profiles. Measurements were conducted in the temperature range 25–950 °C under nitrogen. A dynamic heating rate of 20 °C min^−1^ with a threshold of 3 wt% min^−1^ was adopted.

The high‐resolution‐TGA (HR‐TGA) evidenced that the PKK precursor undergoes a two‐step thermal degradation. The first event at around 331 °C (*T*_I_) is associated with the degradation of the propylene ketone repeat units of the backbone chains, which are responsible for the properties of amorphous domains of materials. The second mass elimination (*T*_II_) is detected at 381 °C and corresponds to the degradation of the polyethylene ketone repeat units of the amorphous domains of polymer backbone chains, while the third event (*T*_III_) is associated with the degradation of the more stable polyethylene ketone repeat units in crystalline domains.^[^ ^8^ ^]^ In addition to the *T*_I_ and *T*_II_ events, P‐FPKK_f_ presents on *f* a further mass elimination (*T*_S_) at a temperature ranging from 150 up to 300 °C (see **Figure** **2**a and Figure S2a, Supporting Information). *T*_S_ is attributed to the degradation of the side chains of (4‐methyl piperazineethyl)]‐N‐ethylene pyrrole repeat units.

The degree of functionalization, *f*, slightly influences *T*_II_, while *T*_I_ and *T*_S_ exhibit a significant dependence on it (Figure S2a, Supporting Information). This is evidence that strong dipole–dipole interactions are present between the side groups of functionalized N‐ethylene pyrrole repeat units, which act to increase the thermal stability of materials. Indeed, *T*_S_ on *f* increases in temperature and decreases in intensity (Figure 2a,c). After quaternization, the membranes in both iodide (P‐FPKK_0.49_(I)_0.45_) and hydroxide (P‐FPKK_0.49_(OH)_0.45_) form show similar profiles (see Figure 2b,d). Notably, the *T*_S_ event occurs in almost the same temperature region as P‐FPKK_f_, thus pointing out that the membrane in quaternized form is thermally stable up to ≈200 °C. The parameters of the above discussed degradation events of materials are plotted in Figure S2, Supporting Information.

The residual mass of the membranes tends to increase on *f* for values lower than *f* = 0.39 (see Figure S2a, Supporting Information) and then decrease, suggesting that the microstructure affects the decomposition process of the material, leading to different amount of char. The lower value of the char with *f* > 0.39 is likely due to the formation at high temperature of a higher concentration of volatile species in the bulk materials with a lower degree of crystallinity.

### 2.3 Thermal Properties Characterization

**Figure 3:**
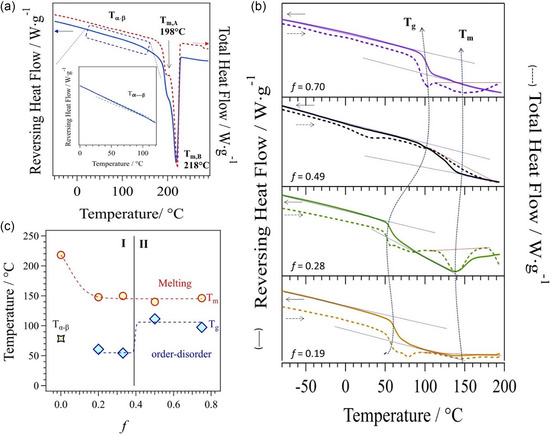
MDSC profiles of: a) pristine polyketone (PKK), the inset shows the *T*_g_ region; b) functionalized P‐FPKK_f_ polyketone at different *f*. c) Dependence on *f* of *T*_g_ and *T*_m_ of P‐FPKK_f_. *f* is the molar fraction of functionalized repeating units (see caption of Scheme 1).

MDSC studies were performed to reveal the types of mesoscale domains of materials and the effect of *f* on their thermal transitions in regions I and II. These studies were conducted in the temperature range from −80 °C to *T*_S_, that is, in the region where, on the basis of HR‐TGA results, the samples are thermally stable. To understand the thermal transitions of materials, it is of crucial importance to carefully study the MDSC profile of the “as‐received” aliphatic PKK precursor terpolymer shown in **Figure** **3**a. The very weak broad secondary band at ≈75 °C, associated with the α → β thermal transition of PKK, demonstrates that small traces of tiny and imperfect α crystalline PKK domains are present in bulk materials. Two distinct primary endothermal events are revealed at ≈198 and 218 °C, attributed to the melting of two types of β crystalline domains of PKK.^[^ ^39^ ^]^ The temperatures of these two thermal transitions coincide with those of a random PKK copolymer,^[^ ^39^ ^]^ along the polymer chain of repeat units of alternating ethylene ketone (E) and propylene ketone (P) in a ratio of 9/1. It should be pointed out that these repeating units along the polymer backbone chains may be assembled in sequences of [E_*i*_PE_*j*_] (A, *i* + *j* = 9) and [E_*k*_PPE_*l*_] (B, *k* + *l* = 18) basic units able to form along the chain axis combination domains as [E_*i*_PE_*j*_]_*m*_[E_*k*_PPE_*l*_]_*n*_ (••AAAA•A_*m*_B_*n*_••).

On this basis, it is to be understood that the two types of crystalline domains associated with the *T*_m,A_ and *T*_m,B_ melting peaks could be attributed to the different distributions of interchain interactions between A and B blocks, respectively. Assuming that the length of each repeating unit along the chain axis is on average 3.8 Å,^[^ ^39^, ^40^, ^41^, ^42^ ^]^ the lengths of the A and B blocks are ≈3.8 and 7.6 nm, respectively. This confirms that traces of very small α crystalline domains present in PKK are responsible for the α → β thermal transition. Likely, α crystalline domains consist of a fraction of very small and compact nanodomains of A blocks interacting with each other. However, *T*_m,A_ and *T*_m,B_ melting peaks likely correspond to β crystalline domains of interacting interchain A and B blocks, respectively.

With respect to PKK, MDSC profiles of P‐FPKK_f_ (Figure 3b) show a completely different behavior. Indeed, there is no α → β thermal event, and two well‐defined thermal transitions, *T*_g_ and *T*_m_, which are detected at *T* < 120 °C and *T* > 140 °C, respectively (Figure 3b,c), are revealed. *T*_m_ is not dependent on *f*, while *T*_g_ exhibits a step change as observed for *T*_I_ (Figure 2a and Figure S2, Supporting Information), thus confirming the presence of the two compositional regions delimited by *f* equal to 0.39, described above. Results show that *T*_g_ is a glass transition event associated with an order–disorder phenomenon of polymer domains based on functionalized chains of B ([E_*k*_PPE_*l*_]_*n*_) domains. It should be noted that in our case (*k* + *l* = 18) the domains based on B repeat units, (*B*)_*n*_ present a higher functionalization degree with respect to those constituted by A repeat units, (*A*)_m_. Indeed, the former could present at maximum '(*k*+*l*)/2' ethylene pyrrole‐functionalized groups per repeating units, while the second '(*i*+*j*‐1)/2' ones. Taken all together, after functionalization of PKK along the backbone chain, domains with two different polarities are formed: 1) polar domains with a high density of functionalized ethylene pyrrole repeat units based on (*A*)_m_ and (*B*)_*n*_ combination domains and 2) hydrophobic domains consisting of a random distribution of close packed and disordered ethylene ketone and propylene ketone repeat units. As expected, after functionalization, the size of hydrophobic domains of the pristine precursor should be dramatically reduced, decreasing the temperature of sharp melting endotherms of PKK from ≈189 to 218 °C to very broad peaks at ≈150 °C in P‐FPKK_f_ (Figure 3b,c). This confirms that a large distribution of very small hydrophobic domains of poly[ethylene ketone/propylene ketone] is present in bulk materials and that pyrrolic repeat units in copolymer chains disrupt the crystalline structure of the pristine PKK terpolymer.^[^ ^34^ ^]^ In addition, these results clearly show that after functionalization no crystalline nanodomains are formed within polar domains of P‐FPKK_f_ due to the presence along the copolymer backbone chains of randomly distributed ethylene pyrrole repeated units.

**Figure 4:**
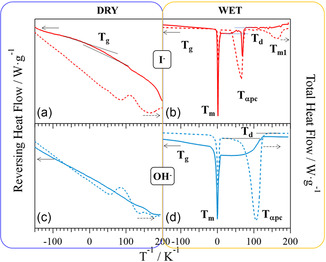
MDSC profiles of quaternized P‐FPKK_0.49_(I)_0.45_ and P‐FPKK_0.49_(OH)_0.45_ in the a,c) dry and in the b,d) wet states. Reversing heat flow (solid lines) and total heat flow (dotted lines), together with primary (*T*_m_, *T*_αpc_, and *T*_m1_) and secondary (*T*_g_ and *T*_d_) thermal transitions, are shown.

Figure 3c demonstrates that the thermal behavior of the two compositional regions of P‐FPKK_f_ are modulated by the *T*_g_ of polar amorphous domains. This shows that in I, the region where a small density of ethylene pyrrole repeated units is present, the degree of order between interacting side chains is lower, thus reducing *T*_g_ down to ≈52 °C, while in II a higher functionalization degree is observed, which increases the strength and the concentration of interchain interactions, raising *T*_g_ up to ≈100 °C. A very weak *T*_g_ is disclosed in the dry P‐FPKK_0.49_(I)_0.45_ membrane at ≈29 °C (**Figure** **4**a), which indicates that polar R_4_N^+^ I^−^ groups of the side chains significantly reduced the order in polar domains of pristine P‐FPKK_0.49_. Therefore, the secondary structure of polymer backbone chains and the interchain interactions between polar side chains are significantly influenced. In dry state, interchain interactions in polar domains of P‐FPKK_0.49_(OH)_0.45_ are further inhibited (Figure 4c), and no *T*_g_ is detected.

In both wet P‐FPKK_0.49_(I)_0.45_ and P‐FPKK_0.49_(OH)_0.45_ membranes (Figure 4b,d), a very weak *T*_g_ at ≈−75 °C is observed, which is not dependent on the type of anion. This prompts us to conclude that no *T*_g_ is present in polar domains due to the plasticizing effect of water and that the higher permittivity of polar domains promotes in hydrophobic domains a small degree of ordering only due to very weak van der Waals interchain interactions. In addition, an endotherm is observed at 0 °C (*T*_f_), which is due to the melting of water in polar nanodomains. A typical transition of AEMs is observed at ≈80 °C, which is associated with the disorder–order transition (i.e., *T*_d_) of wet hydrophilic domains. This event is observed in polar domains when strengthening of electrostatic interactions between the polar side groups takes place as the temperature of the material is raised and the decoupling of water molecule dynamics from polymer relaxation modes occurs. Briefly, as the sample temperature rises, the van der Waals interactions in hydrophobic domains are weakened, facilitating the reciprocal sliding of backbone chains which then promotes the dipole–dipole interactions between polar side chains. This reduces the mobility of I^−^ or OH^−^ ions and decreases the conductivity of the material (see Figure 8b).^[^ ^43^ ^]^

Furthermore, a broad transition at ≈65 and 106 °C is observed (*T*_αpc_) for P‐FPKK_0.49_(I)_0.45_ and P‐FPKK_0.49_(OH)_0.45_ samples, respectively (Figure 4b,d). *T*_αpc_ is the typical thermal transition attributed to the water‐facilitated long‐range relaxation of both backbone and side chains. This transition occurs within hydrophilic domains of the ionomer when the weakening of electrostatic interactions occurs.^[^ ^44^ ^]^

Taken all together TGA and MDSC studies have allowed the study of the thermal stability of materials and shed light on the phenomena modulating their structural properties and interactions in compositional regions I and II.

### 2.4 ATR–FTIR Structural Analysis

ATR–FTIR measurements have been conducted to investigate the structure and the interactions of the proposed materials. ATR–FTIR spectra of PKK, reference compounds, P‐FPKK_f_, and P‐FPKK_0.49_(*X*)_0.45_ are shown in Figure S3–S5, Supporting Information. A correlative assignment of the spectra (**Table** **2**, Figure S3, Supporting Information) is conducted on the basis of vibrational studies of other analogous systems. Particularly, the absorption bands in the region of 2939–2807 cm^−1^ (Figure S3, Supporting Information) are attributed to the antisymmetric and symmetric stretching of –CH_2_ groups of the polar side chains.^[^ ^45^ ^]^ The –NH stretching modes in the region of 3000–3500 cm^−1^ are assigned on the basis of the spectrum of 1‐(2‐aminoethyl)piperazine (A) shown in Figure S3, Supporting Information. As elsewhere described,^[^ ^45^ ^]^ primary amines generally exhibit two peaks while secondary amines only one peak. After the functionalization reaction, P‐FPKK_f_ copolymers showed the band associated with the secondary –NH amine at 3325 cm^−1^ (inset of Figure S3, Supporting Information). No bands attributed to the primary amines of 1‐(2‐aminoethyl)piperazine are evident (Figure S3, Supporting Information and inset). This is evidence that the grafting reaction of piperazine rings to the backbone chain of PKK by Paal–Knorr reactions took place and any unreacted amine was successfully removed during the washing of the polymer. In the PKK spectrum, the intense band at ≈1689 cm^−1^ is ascribed to the stretching mode of the carbonyl groups^[^ ^8^, ^46^ ^]^ of ethylene ketone and propylene ketone repeat units in the crystalline phase. The structural changes occurring on *f* in the copolymer backbone during the synthesis were monitored by measuring the ATR–FTIR spectra of P‐FPKK_f_ (Figure S4, Supporting Information). The results show that increasing the degree of functionalization (*f*) there is an increase in intensity of the peak at ≈1700 cm^−1^ relative to the ν(C=O) mode of the amorphous PKK phase, indicating the disordering effect of the presence of the side functional groups on the polymer chain packing (see right inset in Figure S4, Supporting Information). A higher degree of functionalization corresponds also an overall decrease in intensity of the bands relative to the carbonyl vibrational modes and the increase of those associated with the pyrrolic ones which are formed in the Paal–Knorr reaction (see left inset in Figure S4, Supporting Information).

**Table 2:** ATR–FTIR band assignments of P‐FPKK_0.49_ and its quaternized derivatives in dry state. (Relative intensities of observed bands: vs, very strong; s, strong; m, medium; w, weak; vw, very weak; b, broad; sh, shoulder).

P‐FPKK_0.49_	P‐FPKK_0.49_ (I)_0.45_	P‐FPKK_0.49_ (OH)_0.45_	Assignment	References
3325 vw	–	–	*ν*_s_(NH)	[45]
2933 m	2939 m	2926 m	*ν*_as_(CH)	[45]
2810 m	–	2807 w	*ν*_s_(CH)	[45]
1698 vs	1681 s	1698 vw, sh	*ν*(C=O) amorphous phase	[8, 46]
1640 w	–	1637 w	*ν*(C=O) of Keto form	[8]
1587 w, sh	1581 w, sh	1581 m	*ν*_as_(C=C) of pyrrole ring + ν(C=C) of enol form	[8, 46, 53]
1502 w	1505 vw	1507 vw	*ν*_s_(C=C) of pyrrole ring	[8]
1439 s	1441 w, sh	1456 w	*δ*_s_(CH_2_)	[45]
1406 sh	1404 vw, sh	1403 vw, sh	*δ*(CH_2_)	[8]
1357 m	1358 w	1358 w	*ω*(CH_2_), ν_s_(C_3_N) piper.	[46, 51]
1319 m	–	–	*τ*(CH_2_)	[62]
1299 m	1294 w	1296 w	*δ*(CH_2_) + ν(C‐N)	[8]
1270 sh	1238 w	1261 w	*τ*(CH_2_) + pyrrole ring torsion	[45]
1150 m	1150 m	1150 m	A_u_ piper. skel, ν(C‐N, C‐C)	[46]
1135 s	–	–	*δ*(NH) piper.	[50, 62]
1125 m, sh	1125 m, sh	1125 m, sh	B_u_ piper. skel, ν(C‐N, C‐C)	[46]
–	1092 vw	1093 w	*ω*(CH_2_) + pyrrole ring torsion	[63]
–	1040 w	1040 w	*ν*(C_4_N^+^)	[46]
1012 w	1011 m	1011 m	*ρ*(CH_2_)	[50, 62]
–	965 m	966 m	C_4_N^+^	[43]
–	925 m	926 m	C_4_N^+^	[50, 52, 64]
796 s, sh	798 vw, sh	793 vw	Ring breathing	[50]
755 s	761 s	754 s	*δ*(CH)_oop_ of pyrrole ring	[8]
664 w	662 vw	663 vw	*δ*(CH)_oop_ of pyrrole ring	[8]
590 m	587 vw	573 vw	*δ*_ip_(C=O) and skeletal vibrations	[8]

The conversion of a primary amine into N‐substituted pyrrole moieties is also confirmed by the intensity of ν(C=C) vibrations around ≈1587 cm^−1^^[^ ^8^ ^]^ (Table 2). The –CH_2_ scissoring and wagging modes at ≈1406 and 1357 cm^−1^, respectively, are slightly shifted to high frequencies after functionalization. This suggests that the incorporation of pyrrolic moieties into PKK backbone chains modulates the conformation of the pristine polymer backbone chains.^[^ ^8^, ^47^, ^48^ ^]^ Additionally, as can be confirmed by the FTIR spectrum of 1,2,5‐trimethypyrrole (1,2,5‐TMPyr),^[^ ^8^, ^45^, ^49^ ^]^ the bands at ≈1299 and 755 cm^−1^ correspond to ν(CN) and (C‐H)_oop_ bending modes of pyrrole rings, respectively. A comparison of the spectra of 1‐(2‐aminoethyl)piperazine (Figure S2, Supporting Information) and of P‐FPKK_f_ materials (Figure S3, Supporting Information) allows easily to attribute the peaks associated with the vibrational modes of piperazine rings. Particularly, the bands peaking at ≈1150, 1135, and 1125 cm^−1^ correspond to the ν(C—N, C—C) ring A_u_, δ(NH), and ν(C—N, C—C) ring B_u_ vibrations of piperazine moieties. The absorption band at ≈796 cm^−1^ is ascribed to the ring breathing modes of piperazine.^[^ ^50^ ^]^ In addition, the weak band at ≈1357 cm^−1^ corresponds the C_3_N– stretching mode of the piperazine ring.^[^ ^51^ ^]^ The strong δ(NH) peak of piperazine at 1135 cm^−1^, which is not overlapped onto other bands, is a diagnostic tool for investigating the effect of functionalization on the backbone structure of P‐FPKK_f_ copolymers. The dependence on *f* of the ratio *I*_1141_/*I*_1689_, where *I*_1141_ and *I*_1689_ are the intensities of δ(NH) and ν(C=O) vibrational modes, respectively, is shown in the inset of Figure S4, Supporting Information. These results are in accordance with the behaviors of WU% and *λ* versus IEC (Figure 1c) and *T*_g_ versus *f* (Figure 3c), thus confirming that two structural regions (I, II) characterize the physicochemical properties of these materials. In region I, it is likely that the strong interchain interactions existent between piperazine side rings increase the symmetry of interacting side chains, reducing the intensity of vibrational modes associated with piperazine rings. In region II, the opposite behavior is expected whereby the disorder between the backbone chains increases and subsequently the intensity of vibrational modes associated with the piperazine rings.

Upon methylation of P‐FPKK_f_ copolymers, P‐FPKK_0.49_(I)_0.45_ ionomer (Figure S5, Supporting Information) shows: 1) two new bands at ≈922 and 965 cm^−1^, attributed to the vibrational modes of quaternary ammonium groups, thus observing a successful methylation of piperazine moieties;^[^ ^46^, ^52^ ^]^ and 2) that the shape and intensity of ν(C—N, C—C) ring A_u_, δ(NH), and ν(C—N, C—C) ring B_u_ vibrations of piperazine rings changed significantly (inset of Figure S5, Supporting Information and Table 2). Concurrently, with respect to P‐FPKK_0.49_, in P‐FPKK_0.49_(*X*)_0.45_ the intensity of the δ(NH) mode peaking at 1135 cm^−1^ significantly decreases.

In P‐FPKK_0.49_(*X*)_0.45_, the band at ≈1581 cm^−1^, is attributed to the superposition of the *ν*_as_(C=C) mode of pyrrole rings^[^ ^8^, ^53^ ^]^ and of ν(C=C) vibrational modes of a fraction of the enol form (Table 2 and Figure S5, Supporting Information) of the PKK moiety. As expected, this latter mode is very broad and gradually increases in intensity as X changes from I^−^ to OH^−^ in the membrane. This observation suggests that in P‐FPKK_0.49_ (OH)_0.45_, the hydrogen bonding network contributes to better delocalizing the charge carriers along the backbone chains. This is confirmed by BES ion conductivity measurements (see Section 2.7).

### 2.5 Nuclear Magnetic Resonance Spectroscopy Studies

**Figure 5:**
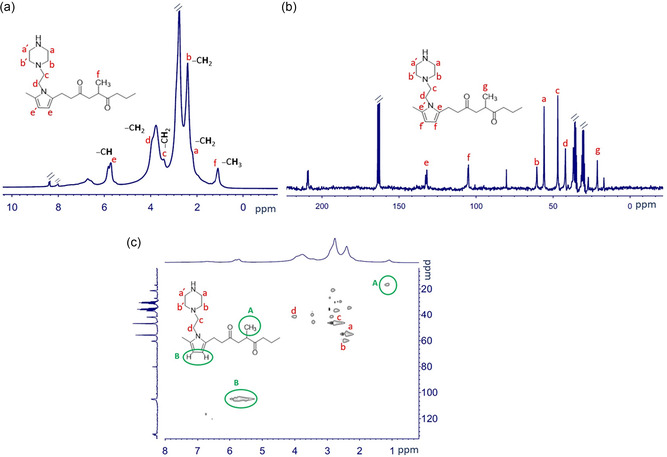
NMR spectra of P‐FPKK_f_ with *f* = 0.49: a) ^1^H NMR; b) ^13^C NMR; c) ^1^H–^13^C HSQC NMR.

The nuclear magnetic resonance spectroscopy analysis proved to be useful in understanding the structure of P‐FPKK_f_ and P‐FPKK_f_(*X*)_g_. **Figure** **5**a,b shows the ^1^H NMR and ^13^C NMR spectra of the polyketone functionalized with 1‐(2‐aminoethylpiperazine) P‐FPKK_0.49_. Since the spectra contain many overlapping peaks, intensities for unreacted ketonic groups are not assigned for brevity. The resonances at 5.75 and 105.52 ppm are assigned to the hydrogen and carbon atoms of the pyrrole ring, respectively, in accordance with the literature.^[^ ^8^ ^]^ The peaks at 46.74 and 42.56 ppm in the ^13^C NMR spectrum and at 3.94 and 3.44 ppm in the ^1^H NMR spectrum are associated with the carbon and hydrogen atoms of the ethylene bridge, respectively.

The resonances corresponding to the piperazine ring atoms are observed at 56.35 and 61.06 ppm in the ^13^C NMR spectrum, and 2.20 and 2.41 ppm in the proton spectrum, indicating a successful functionalization of polyketone. In addition, to ensure the structural assignment of P‐FPKK_0.49_, a 2D ^1^H–^13^C HSQC was performed. The peaks corresponding to the carbon of the methyl group of PKK domains correlate to the protons at 1.12 ppm (Figure 5c), ensuring that the propylene groups of the original PKK are not involved in the Paal–Knorr ring closure reaction due to the steric effect it offers to cyclization.^[^ ^36^ ^]^ The carbon atom of the methyl group on the pyrrole is expected to have traces at around 22 ppm, which are absent in this case; therefore, the preferential site for the cyclization is the ethylene diketone unit (Scheme 1).

^1^H–^13^C HSQC was also used to determine the site of quaternization, which in P‐FPKK_0.49_(I)_0.45_ was confirmed to be the piperazine nitrogen bound to the ethylene bridge. Thus, the cationic side groups are 1,4‐dimethyl piperaziniumethyl ones (see Supporting Information and Figure S1, Supporting Information).

### 2.6 X‐Ray Diffraction Studies

**Figure 6:**
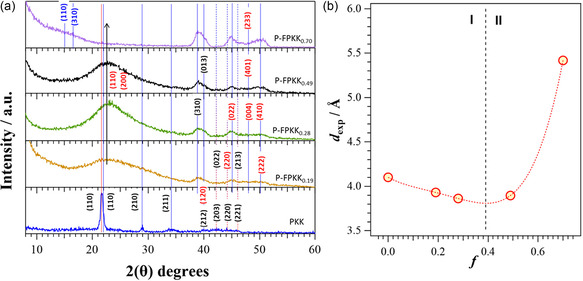
a) X‐ray diffraction pattern of PKK and P‐FPKK_f_ samples at different *f* values. b) Dependence on *f* of interplanar distance *d*_(110)_ of β phases. Regions I and II are shown delimited by *f* = 0.39. The (*hkl*) indexes reported in black are assigned based on DFT minimizations of starting α and β structural parameters of ref. [40, 41], in blue by minimizing the structural parameters reported in ref. [42], and in red by simply calculating the WAXD patterns on the basis of the structural parameters reported in ref. [42]

These studies are conducted with the aim to shed light on the structure of the crystalline and amorphous domains of the proposed materials. **Figure** **6** shows the X‐Ray diffraction (XRD) pattern of PKK and P‐FPKK_f_ at various *f*. The PKK pattern exhibits features which are typically attributed to the α and β polyketone phases.^[^ ^39^ ^]^ In particular, peaks measured at 2θ ≈ 21.8°, 29°, 34°, and 42° correspond to the (110), (210), (211), and (203) reflexes of β phase, respectively, while the low‐intensity peaks at 2*θ* = 21.6° (shoulder) and 40° correspond to the (110) and (022) reflexes of the α phase, respectively. Results show that PKK consists of crystalline domains of poly(ethylene ketone/propylene ketone) mainly composed of β phases blended with small traces of α phases (**Table** **3**). The assignment of the PKK spectrum (Figure 6) is confirmed by calculating the diffraction pattern (see Experimental Section) of this copolymer in α and β forms by adopting the unit cell parameters, the fractional coordinates, and the space groups *Pnma* (no. 62) and *Pbnm* (no. 62) elsewhere reported,^[^ ^39^, ^41^, ^42^ ^]^ respectively. Table 3 summarizes the 2*θ* peaks of the spectra of PKK and of selected P‐FPKK_f_ (*f* = 0.49 and 0.70) samples shown in Figure 6a.

**Table 3:** Assignments of 2*θ* peaks and corresponding interplanar distances measured by WAXD patterns of PKK and P‐FPKK_f_ (*f* = 0.49 and 0.70). Miller (*hkl*) indexing of spectra is carried out using the calculated spectra of α and β phases determined on the basis of crystallographic data reported in ref. [41, 42]. Calculated WAXD patterns of PKK and P‐FPKK_f_ are obtained as described in the Experimental Section. A) Values in black are determined by DFT minimizations of starting α and β structural parameters’ reported values in refs. [40, 41]. b) Values in blue are obtained by minimizing the structural parameters reported in ref. [42] and c) values in red by simply calculating the WAXD patterns on the basis of the structural parameters reported in ref. [42].

PKK α^(calc)^ β^(calc)^				P‐FPKK_0.49_ β^(calc)^			P‐FPKK_0.70_ β^(calc)^		
2*θ*_exp_	*d*_exp_ [Å]	*d*_c_/Å [*hkl*]	*d*_c_/Å [*hkl*]	2*θ*_exp_	*d*_exp_ [Å]	*d*_c_/Å [*hkl*]	2*θ*_exp_	*d*_exp_ [Å]	*d*_c_/Å [*hkl*]
–	–	–	–	–	–	–	15.25 (w)	5.80	5.68 (110)
–	–	–	–	–	–	–	16.35 (w)	5.41	5.38 (310)
21.65 (sh)	4.10	4.11 (110)	–	–	–	–	–	–	–
21.8 (s)	4.07	–	4.09 (110)	22.8 (s)	3.90	3.99 (200) 3.79 (110)	–	–	–
22.25 (w)	3.99	3.80 (002)	3.79 (002)	–	–	–	–	–	–
28.95 (w)	3.08	–	3.09 (210)	–	–	–	–	–	–
34.25 (vw)	2.62	–	2.83 (211)	–	–	–	–	–	–
39.7 (vw)	2.27	2.29 (212)	2.28 (120)	39.0 (w)	2.31	2.30 (310)	39.1 (s)	2.30	2.28 (120)
–	–	–	–	40.4 (w)	2.23	2.23 (013)	40.45 (m,sh)	2.23	2.23 (013)
42.3 (vw)	2.13	2.12 (022)	2.13 (203)	–	–	–	–	–	–
44.0 (vw)	2.06	2.06 (220)	2.04 (220)	–	–	–	–	–	–
–	–	–	–	45.0 (w)	2.01	2.01 (022)	44.95 (s)	2.01	2.01 (022)
45.9 (vw)	1.97	1.98 (221)	1.95 (213)	–	–	–	–	–	–
–	–	–	–	47.45 (vw)	1.91	1.93 (401) 1.89 (004)	46.75 (m)	1.94	1.94 (233)
–	–	–	–	–	–	–	48.75 (m)	1.87	1.87 (114)
–	–	–	–	49.8 (w)	1.83	1.84 (410)	49.95 (m)	1.82	1.84 (410) 1.82 (222)

The Miller indexing of the reflexes is shown in Table 3 and Figure 6a. Specifically, in the figures of P‐FPKK_f_ spectra with color: 1) black corresponds to values determined by density functional theory (DFT) minimizations from α and β structural parameters reported in ref. [40, 41]; 2) blue is obtained by minimizing the structural parameters reported in ref. [42]; and 3) red is calculated from the WAXD patterns on the basis of the structural parameters reported in ref. [42]

Summarizing, results demonstrate that: 1) PKK copolymer consists mainly of a β‐phase doped with traces of α phases. This outcome is in accordance with DSC results; and 2) after PKK functionalization, the (110) and (310) peaks of the β‐phase broaden and shift to lower 2*θ*. This indicates that the introduction of functionalized ethylene pyrrole repeat units along the backbone chains acts to increase the rotational disorder in the plane normal to the *c*‐axis, affecting the length of the *a*‐ and *b*‐axis and the distances between the copolymer chains. No significant changes of the interplanar distance along the c‐axis were observed, that is, along the axis of polymer backbone chains.

The dependence on *f* of *d*_(110)_ of P‐FPKK_f_ samples (Figure 6b) shows the similar behavior of WU% and *λ* versus IEC (Figure 1c) and *T*_g_ versus *f* (Figure 3c). This demonstrates that at *f* ≤0.39, the *d*_(110)_ interchain spacing slightly decreases, improving the close packing of P‐FPKK_f_ chains (region I), decreasing significantly the size of the crystalline domain (peaks become broader and decrease in intensity). However, at *f* ≥ 0.39, the opposite behavior is observed revealing that in region II interchain interactions are weakened and the interchain free volume of materials is increasing on *f*.

In summary, the introduction of ethylene pyrrole repeat units in copolymer backbone chains yields P‐FPKK_f_ samples with β phases and modulates significantly the degree of crystallinity of the resulting functionalized copolymers. Indeed, as *f* increases, the amount of amorphous domains (rotational disorder of P‐FPKK_f_ chains) increases and the size of the crystalline domains is significantly reduced. This morphological modulation of copolymer domains is likely the result of the weakening of the interchain carbonyl interactions of the pristine copolymer due to the increasing density of ethylene pyrrole repeat units along the P‐FPKK_f_ chains.

### 2.7 Conductivity and Conduction Mechanism Studies

**Figure 7:**
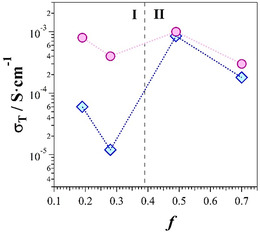
Dependence on *f* of ionic conductivity (*σ*_T_) measured at room temperature of membranes P‐FPKK_f_(I)_g_ and P‐FPKK_f_(OH)_g_, respectively. The P‐FPKK_0.49_(*X*)_0.45_ membranes exhibit the highest value of ionic conductivity at room temperature. Regions I and II delimited by *f* = 0.39 are shown.

**Figure 8:**
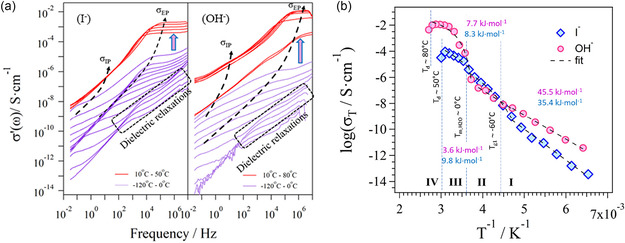
a) 2D profile of the real component of conductivity, *σ*′(*ω*), as a function of frequency of wet FPKK_0.49_(*X*)_0.45_ membranes in iodide (I^−^) and hydroxide (OH^−^) form. b) Dependence of log(*σ*_T_) on 1/T for P‐FPKK_0.49_(X)_0.45_ with X = I^−^ and OH^−^. Four conductivity regions (I, II, III, and IV) are shown which are delimited by the thermal transitions detected by MSDC (*T*_g_, *T*_m_, and *T*_δ_). The dashed lines represent the fitting curves. These are Arrhenius like in region I and VTF like in regions II and III.

The wet P‐FPKK_f_(*X*)_g_ membranes in iodide and hydroxide form were analyzed by BES to detect the polarization and dielectric relaxation phenomena characterizing the electric response of these materials. **Figure** **7** shows the profile of the dependence on *f* on the ionic conductivity (*σ*_T_) of P‐FPKK_f_(*X*)_g_ at room temperature, which exhibits the same behavior as WU% and *λ* versus IEC (Figure 1c) and *T*_g_ versus *f* (Figure 3c) in both forms. This indicates that the conductivity mechanism of P‐FPKK_f_(*X*)_g_ materials is strongly correlated to the structural features of polymer hosting matrices. Due to its highest conductivity value (0.99 mS cm^−1^ at RT, Figure 7), P‐FPKK_0.49_(*X*)_0.45_ material was selected with X = I^−^ and OH^−^ in order to carry out a detailed study of the electric response as a function of frequency (0.03–10^7^ Hz) and temperature (−120–80 °C). The 2D curves of the real component of complex conductivity, *σ′*(*ω*), as a function of frequency of wet P‐FPKK_0.49_(*X*)_g_ materials in iodide and hydroxide form are shown in **Figure** **8**a. Results show that the electric response on frequency is dominated by two polarization phenomena. Indeed, in the low‐frequency wing, a short plateau is revealed which corresponds to an interdomain polarization event (*σ*_IP_). This is better defined for the membrane in OH^−^ form. In the high‐frequency wing, a plateau is detected (Figure 8) that corresponds to the electrode polarization phenomenon (*σ*_EP_).

In the membrane, in hydroxyl form, *σ*_EP_ is more intense and shifts to higher frequencies, thus indicating that in hydrophilic domains of P‐FPKK_0.49_(OH)_0.45_, OH^−^ exhibits a mobility and a delocalization higher than I^−^.

In addition, it is revealed that in correspondence to the melting temperature of water domains (0 °C) a step rising of *σ′*(*ω*) at frequencies higher than 10^4^ Hz is revealed, which is approximately two and three orders of magnitude in the membrane in I^−^ and OH^−^ form, respectively. This suggests that the clusters of water embedded in hydrophilic domains of bulk membranes increase in size in the order P‐FPKK_0.49_(I)_0.45_ ≪ P‐FPKK_0.49_(OH)_0.45_. Furthermore, the peaks measured in the σ′(ω) profiles of materials (Figure 8a) at frequencies ranging from 10^2^ to 10^7^ Hz and at temperatures lower than −80 °C are attributed to the α‐ and β‐dielectric relaxations of the copolymer matrix. In accordance with other studies,^[^ ^8^, ^18^, ^33^, ^54^ ^]^ interdomain polarizations (*σ*_IP_) are attributed to the accumulation of charge at the interfaces between domains with different permittivities in bulk membranes. In general, this event is diagnostic of the heterogeneities of materials at the mesoscale.^[^ ^8^, ^18^, ^33^, ^54^ ^]^

The electrode polarization (*σ*_EP_) is assigned to the accumulation of charge at the interface between the electrode and the membrane.^[^ ^18^ ^]^ *σ*_IP_ of the membrane in OH^−^ form is more intense and better defined than that of the membrane in I^−^ form, thus indicating that the dissociation of OH^−^ anions in the membrane is higher than that of iodide.

For membranes in both iodide and hydroxide form, *σ*_EP_ ≫ *σ*_IP_ by at least three orders of magnitude. This demonstrates that the overall conductivity (*σ*_T_) of materials, which in P‐FPKK_0.49_(*X*)_0.45_ (*X* = I^−^, OH^−^) is the superposition of both the *σ*_EP_ and *σ*_IP_ contributions (*σ*_T_ = *σ*_EP_ + *σ*_IP_), coincides with *σ*_EP_ (*σ*_T_ ≈ *σ*_EP_). This allows us to easily determine *σ*_T_ in the midfrequency region of *σ*_EP_ plateau. The dependence of *σ*_EP_ on 1/T of P‐FPKK_0.49_(*X*)_0.45_ membrane in I^−^ and OH^−^ is shown in Figure 8b. Four conductivity regions are detected which are delimited by the thermal transitions determined by MDSC (*T*_g_, *T*_m_, and *T*_d_). *T*_d_ is detected at ≈60 and 97 °C for the membranes in iodide and hydroxyl forms, respectively, and corresponds to a disorder–order transition.^[^ ^18^ ^]^ In general, this latter event occurs in membranes with polar side chains characterized by weakly dissociated anions. Thus, in the head of side chains, dipoles are formed along the backbone polymer. These dipoles give rise to strong interchain electrostatic interactions which inhibit the mobility of the anions, thereby decreasing the conductivity of the materials. Results demonstrate that in membranes: 1) I^−^ is less dissociated than OH^−^ and 2) the mobility and the delocalization of anions in hydrophilic domains rises in the order I^−^ < OH^−^. This is also confirmed by the maximum conductivity value of P‐FPKK_0.49_(*X*)_0.45_ membranes which at 50 °C (*X* = I^−^) and 80 °C (*X* = OH^−^) exhibit 2.05 and 11.7 mS cm^−1^, respectively.

The low conductivity could be attributed to cation clustering. In general, AEMs contain positively charged groups interacting with the negative counterion which contribute to the ionic conductivity of the hydrated membrane. Depending on the mesoscale microstructure of the polymer in the membrane, these groups interact strongly with each other giving rise to clustering. When the membrane absorbs water, these cationic groups become more mobile and interact more easily, particularly at high temperatures. This phenomenon constrains the extent to which a membrane's conductivity can be improved, even if more charged groups are added (high IEC).^[^ ^43^ ^]^ In our case, both the order–disorder phenomena (*T*_g_) and hydration properties (WU% and *λ*), which contribute to the ion conductivity, have proved to strongly depend on the *f* and the consequent conformational geometry assumed by the polymer backbone and the interchain interactions between the side polar groups.

In region I, at *T*_g_ < −60 °C, Arrhenius‐like behaviors, with an activation energy (*E*_a_) of 35.4 and 45.5 kJ mol^−1^, are shown for P‐FPKK_0.49_(*X*)_0.45_ in iodide and hydroxide form, respectively. In this temperature region, the high *E*_a_ value suggests that along *σ*_IEP_ conductivity pathways,^[^ ^18^, ^33^, ^43^ ^]^ long‐range charge migration processes are mainly modulated by hopping processes of anions between coordination sites. In regions II and III, both membranes show a Vogel–Tamman–Fulcher (VTF)–like behavior^[^ ^33^ ^]^ with an *E*_a_ of ≈3–9 kJ mol^−1^. Therefore, the segmental motion of the polymer backbone chains based on poly(ethylene pyrrole) repeat units is crucial in modulating long‐range charge migration events in bulk membranes.

### 2.8 Alkaline Stability of P‐FPKK_0.49_(OH)_0.45_ Membrane

One of the most challenging targets to achieve today in AEMs is to improve their long‐term stability in alkaline conditions. This requires that both the side and the backbone polymer chains of the ionomers are able to withstand the extreme pH conditions present in alkaline fuel cells without relevant chemical degradation. The most common degradation process of AEMs occurs either by S_N_2 substitution mechanisms, Hoffmann elimination, or hydroxy group formation.^[^ ^13^ ^]^ In recent years, great effort has been devoted by researchers to understand the factors responsible for the degradation processes and to increase membrane stability. This was performed by modulating the basicity, the steric hindrance, and the electronic configuration of the polar side groups together with other types of external factors such as temperature, solvents, and others.^[^ ^55^, ^56^, ^57^ ^]^

Six‐membered cycloaliphatic quaternary ammonium groups, such as N,N‐dimethylpiperidinium, 5‐azonia‐spiro[4.4]nonane, and 1,4‐diazabicyclo[2.2.2]octane, exhibit a higher alkaline stability against elimination and ring‐opening substitution processes due to their antiperiplanar β protons with —C—C— bonds rotationally protected by the ring geometry. Briefly, the low ring strain and the increased transition state energy of degradation processes, resulting from the chemical stability of their bond angles and lengths, contribute significantly to the enhancement of membrane durability in alkaline conditions.^[^ ^55^ ^]^ Here, the alkaline stability of the piperazinium‐modified PKK membranes is studied over time by BES and vibrational spectroscopy, before and after immersion of membrane into a 1 m KOH (aq.) solution at 60 °C. During the durability experiment, which lasted two weeks, the color of the membrane changed from an initial dark brown color to a final light brown one.

**Figure 9:**
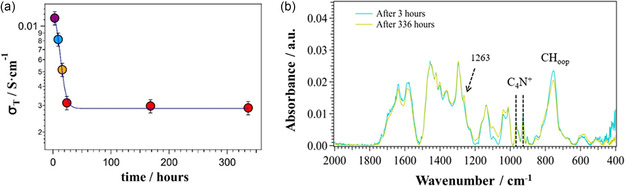
a) Dependence of the overall conductivity (*σ*_T_) of P‐FPKK_0.49_(OH)_0.45_ on time. *σ*_T_ is determined by monitoring the ion conductivity of the membrane immersed in 1 m KOH solution at 60 °C over a period of 336 h. b) ATR–FTIR spectra of the membrane after aging in an alkaline solution (1 m KOH (aq.)) at 60 °C for 3 and 336 h. The spectra are normalized to the peak at 1135 cm^−1^.

The dependence on time of *σ*_T_ (**Figure** **9**a) shows that the conductivity of P‐FPKK_0.49_(OH)_0.45_ decreases sharply in the first 24 h and then stabilizes for the remaining 312 h at ≈25% of its initial *σ*_T_ value (11.3 mS cm^−1^).

A comparison of the ATR–FTIR spectra of membranes before and after aging for 336 h (Figure 9b) shows that no significant changes are revealed by the vibrational modes of the quaternary ammonium side groups and by the copolymer backbone chains. A slight increase in the intensity of the weak band at ≈1263 cm^−1^ is observed which is attributed to the CH_2_ rocking vibrational mode and to the antisymmetric ν(CCN) stretching vibrations of tertiary amines.^[^ ^45^ ^]^ This is evidence of a partial degradation of the piperazinium cations after 336 h of aging, probably associated with a nucleophilic attack by the hydroxide ion on the quaternized polar ammonium groups.^[^ ^55^ ^]^ Based on the observed influence of the degree of functionalization and quaternization on the microstructure of the polymers, it is plausible that some ion exchange groups are present in less accessible domains within the bulk of the material, thus contributing less to the overall conductivity in the pristine material, but also less affected by the high concentration of OH^−^ in the solution. These groups can contribute to the low but stable ion conductivity observed after the initial drop.

The other bands associated with the vibrational modes of the polymer backbone at 1638, 1296, and 754 cm^−1^ are unaffected by the alkaline environment, with only a slight decrease in intensity of the band at around 1581 cm^−1^, which corresponds to the *ν*_as_(C=C) mode of the pyrrole ring. These results confirm that after 336 h of aging in the above aggressive alkaline conditions, the ionomer still maintained its structural integrity. This classifies the P‐FPKK_0.49_(OH)_0.45_ membrane as a promising material for application in alkaline fuel cells or as water electrolyzers.

## 3 Conclusion

This report describes the preparation and the study of P‐FPKK_f_ precursors and P‐FPKK_f_(*X*)_g_ AEMs with *X* = I^−^ and OH^−^. P‐FPKK_f_ precursors are prepared by the functionalization of polyketone (PKK) with 1‐(2‐aminoethyl) piperazine by means of a Paal–Knorr reaction. P‐FPKK_f_(*X*)_g_ are obtained by the quaternization of P‐FPKK_f_ precursors, yielding materials potentially alternative to the existing expensive alkaline ionomers used today for application in alkaline fuel cells or electrolyzers. The attractiveness of this synthetic route lies in the high degree of tunability of both the properties and structure of the resulting membranes. A systematic investigation of the properties and structure of the prepared materials was conducted by HR‐TGA, MDSC, XRD studies, and ATR–FTIR and NMR spectroscopies. Results of the FPKK_f_(*X*)_g_ studies revealed that: 1) the IEC is linearly dependent on the degree of functionalization *f*; 2) the thermal stability of FPKK_f_(*X*)_g_ ionomers extends up to ≈200 °C; and 3) two compositional regions, I and II, delimited by *f* = 0.39, are present in the d_(110)_ versus *f* (Figure 6b), WU% and *λ* versus IEC (Figure 1c), and *T*_g_ versus *f* (Figure 3c) profiles. This demonstrates that the functionalization of PKK is very effective and that in ionomers with *f* ≤ 0.39, a higher density of interchain dipole–dipole interactions exists along the polymer chains between ethylene ketone repeat units that tightly compact the ionomer, reducing the values of its WU%, *d*_(110)_, and *λ*. At *f* > 0.39, the higher density of functionalized ethylene pyrrole repeat units along the polymer chain axis surpasses the concentration of ethylene ketone repeating units. This disrupts the strong network of interchain interactions between ethylene ketone repeat units and promotes the interchain interactions between the bulky 1,4‐dimethylpiperaziniumethyl side chains increasing the WU%, *d*_(110)_, *λ*, and the *T*_g_ of materials.

Taken all together, these studies allowed us to conclude that in ionomers with a high degree of functionalization, *f* > 0.39, the interchain distance, interactions, and free volume between 1,4‐dimethylpiperaziniumethyl side chains increase significantly with respect to the materials at *f* ≤ 0.39, thus significantly modulating the physicochemical properties of the materials.

In addition, multinuclear correlation NMR study demonstrated that the Paal–Knorr reaction is highly effective and selective toward the functionalization of poly(ethylene ketone/propylene ketone) backbone chains. Indeed, only vicinal ketone groups of the ethylene ketone repeat units are able to give rise to Paal–Knorr reactions. No evidence of the involvement of propylene ketone repeat units in the Paal–Knorr reaction is revealed.

BES studies of P‐FPKK_0.49_(OH)_0.45_ membranes showed a water uptake of 43% and at 60 °C a conductivity of ≈11.3 mS cm^−1^. Finally, it should be highlighted that grafting of the aliphatic six‐membered piperazinium cation side chains onto the N‐pyrrole ethylene repeat units of the backbone chain provides membranes with excellent chemical stability over the period of 336 h in 1 m KOH solution at 60 °C. In conclusion, we can safely state that the obtained results concur to demonstrate that the membranes proposed here are promising candidates for testing in cell conditions in view of a possible application in alkaline electrochemical energy conversion and storage devices.

## 4 Experimental Section

4.1

4.1.1

##### Materials

Polyketone PK930 (PKK) was provided by Hyosung Co., Ltd (Seoul Korea). 1‐(2‐aminoethyl)piperazine *M*_w_ = 129.20 (g mol^−1^), *m*‐cresol (CH_3_C_6_H_4_(OH)), chloroform (CHCl_3_), diethyl ether (C_2_H_5_O), iodomethane (CH_3_I), dimethylformamide (DMF, C_3_H_7_NO), and potassium hydroxide (KOH) were purchased from Sigma Aldrich. All reagents and solvents were used as received without any further purification.

##### Synthesis of P‐FPKK_f_

Polyketone (PKK) precursor was reacted with 1‐(2‐aminoethyl)piperazine (A) for 48 h in *m*‐cresol, (step 1 of Scheme 1), according to a protocol previously reported.^[^ ^32^ ^]^ To study the effect of the degree of functionalization on the physicochemical properties of membranes, the synthesis was performed at different amine(A)/(1,4‐diketonic repeat units) (PKK) molar ratios: A/PKK = 0.25/1, 0.5/1, 1/1, and 3/1. Depending on A/PKK molar ratios, the reaction mixture consistency changes from that of a light brown liquid solution with a low viscosity, to that of a highly viscous dark brown honey‐like solution. After reaction, the resulting mixture was dissolved in chloroform and the polymer precipitated with diethyl ether. This procedure was repeated at least three times to eliminate the excess of reagent. The obtained product was dried at 60 °C overnight and then kept in an N_2_ glovebox at 80 °C for 24 h inside a cold finger under a vacuum of 10^−2^ mbar.

##### Membrane Fabrications

A homogeneous solution of the polymer was obtained by stirring 300 mg of P‐FPKK_f_ powder for 5 h in 25 mL of DMF. To obtain a membrane, this was first microfiltered and then cast onto a Teflon Petri dish at 60 °C overnight. The membrane was detached from the Petri dish by immersion in water, then dried in a vacuum desiccator for 24 h and finally placed into a N_2_ glovebox inside a cold finger at 80 °C. The thickness of the membrane ranged from 50 to 70 μm.

##### Quaternization of P‐FPKK_f_(I)_g_

The dry membrane was immersed in an excess of neat iodomethane (CH_3_I) solution for 24 h. The excess of iodomethane was removed by washing the membrane with diethyl ether and finally with double‐distilled water for at least three times. The membrane was then dried in a desiccator for 24 h under an N_2_ flow and then inside an N_2_ glovebox for 5 days in a cold finger at 10^−2^ mbar and 60 °C (dry membrane). A fully hydrated membrane ('the wet membrane') for conductivity measurements was obtained by immersion of the membrane in double‐distilled water for 48 h at room temperature.

##### P‐FPKK_f_(OH)_g_ Membrane

The membrane in hydroxyl form (P‐FPKK_f_(OH)_g_) was obtained by immersing P‐FPKK_f_(I)_g_ in a 1 m KOH solution, inside a CO_2_‐free glovebox for 1 h. This process was repeated three times, each time using a fresh KOH solution. The resulting membrane was finally washed three times with degassed double‐distilled water to remove any remaining KI and then stored in a hermetically sealed bag in double‐distilled water. By drying the membrane in an argon‐filled glovebox at 70 °C under vacuum for 3 h, the dry samples were obtained.

##### Structure and Compositions

ATR‐FTIR spectra were collected in the midinfrared range (4000–400 cm^−1^) using a Nicolet Nexus spectrometer. Spectra were obtained using a single‐beam ATR measuring cell with a diamond crystal sealed under inert atmosphere (Specac MKII Golden Gate ATR System GS10563). Spectra were the result of averaging 1000 scans. The ATR cell was prepared and sealed inside a glove box under argon atmosphere.

Multinuclear NMR experiments were measured using a 400 MHz Bruker Avance spectrometer by dissolving the sample in deuterated dimethylformamide (DMF‐d6).

Composition of samples was determined by elemental analyses (CHNS/O) using a FLASH‐2000 CHNS/O Organic Elemental Analyzer (Thermo Fisher Scientific).

##### Thermal Stability Measurement

HR‐TGA were conducted in a temperature range of 25–950 °C under a nitrogen flow of 100 cm^3^ min^−1^ using a high‐resolution Thermobalance (TA Instruments 2950). Measurements were performed on ≈5–10 mg of the sample. A heating rate of 20 °C min^−1^ in dynamic heating rate mode with a threshold of 3% min^−1^ was adopted. The resolution of the thermobalance was 0.1 μg.

MDSC analyses were carried out using a TA Instruments DSC Q20 calorimeter equipped with a liquid nitrogen heating–cooling system. Measurements were obtained in a temperature range from −150 to 200 °C on ≈8–10 mg of sample, which was loaded inside of a hermetically sealed aluminum pan inside an argon glove box.

##### XRD Measurements and Assignments

X‐ray diffraction patterns were measured in transmission mode using an eXplorer diffractometer (GNR Analytical instruments) operating with a monochromatized Cu Kα radiation (*λ* = 1.5406 Å). The spectra were measured in a 2*θ* range 8–90° with a 0.5° step, with a Bragg–Brentano geometry and an integration time of 5 min.

The assignments of WAXD spectra of PKK in α and β forms were obtained by adopting the unit cell parameters, the fractional coordinates, and the space groups *Pnma* (no. 62) and *Pbnm* (no. 62), elsewhere reported,^[^ ^41^, ^42^ ^]^ respectively. The assignment of P‐FPKK_f_ WAXD spectra was obtained using the WAXD spectra calculated by means of the results of energy minimization of the structures of P‐FPKK_f_ materials. These computations were performed using as starting parameters the structural features of α and β phases of PKK reported in literature and the DFT methods implemented in an all‐electron DFT code using the DMol3 program^[^ ^58^, ^59^ ^]^ as a part of the Materials Studio package (double numerical plus polarization basis set, gradient‐corrected (GGA) BLYP functional).

##### Broadband Electrical Spectroscopy (BES)

The electric response of wet membranes was measured in the frequency and temperature range from 0.03 to 10^7^ Hz and –120 °C to *T*_max_, respectively (*T*_max_ was 60 or 80 °C for the membrane in I^−^ or OH^−^ form, respectively). Measurements were performed using a Novocontrol Alpha‐A analyzer with a potential amplitude of 0.1 V. The temperature was raised by 10 °C/step using a homemade heating–cooling system operating with liquid N_2_. The temperature was measured with an accuracy better than ±0.2 °C. Before measurements, the sample was soaked overnight in double‐distilled water and then sandwiched between two platinum electrodes inserted inside a sealed measuring cell. No correction for the thermal expansion of the cell was adopted.

##### Ion Exchange Capacity (IEC)

IEC of the P‐FPKK_f_(I)_g_ membranes was determined by Mohr titration of the anion^[^ ^60^ ^]^ as follows: 1) I^−^ of P‐FPKK_f_(I)_g_ was exchanged with Cl^−^ by soaking the membrane in 1 m aqueous KCl solution for 48 h; 2) the membrane in Cl^−^ form was washed repeatedly every 2 h with double‐distilled water for 12 h in order to remove all traces of Cl^−^ and K^+^ ions; and 3) the obtained membrane was then immersed in a 0.1 m aqueous NaNO_3_ solution for 48 h to exchange Cl^−^ with NO_3_^−^ ions. To ensure that the Cl^−^ anion was completely exchanged with NO_3_^−^, the NaNO_3_ solution was renewed three times every 12 h. The collected solutions were titrated with a 0.01 m aqueous AgNO_3_ solution ($C_{{\text{AgNO}}_{3}}$), using a few drops of an aqueous K_2_CrO_4_ solution (0.1 m) as a colorimetric indicator. After titration, the membrane was dried for 48 h under vacuum and weighed. The IEC was determined from the volume of consumed AgNO_3_ ($V_{{\text{AgNO}}_{3}}\;$with $C_{{\text{AgNO}}_{3}}$ concentration) and from the mass of the dry membrane (*W*_dry_).

(4) $$\text{IEC}({\text{meq g}}^{-1})=\frac{\left(V_{{\text{AgNO}}_{3}})(C_{{\text{AgNO}}_{3}}\right)}{W_{\text{dry}}}$$

The IEC of the membrane in hydroxyl form was determined using a back titration method^[^ ^61^ ^]^ as follows: 1) the membrane was first immersed in a standard HCl solution (0.05 m, 30 mL) for 48 h at room temperature to neutralize the OH^−^ ions and 2) the resulting solution was then titrated with a 0.05 m NaOH standard solution using phenolphthalein as an indicator. The membrane was then: 1) washed and immersed in deionized water for 24 h to remove the residual HCl; 2) dried under a vacuum of 10^−2^ mbar at 60 °C for 24 h; and 3) finally weighed to accurately determine the mass of the dry sample. The IEC was determined by the following equation

(5) $$\text{IEC}({\text{meq g}}^{-1})=\frac{n_{\left(i,H^{+}\right)}-n_{\left({f,H}^{+}\right)}}{W_{\text{dry}}}$$

where $n_{\left(i,H^{+}\right)}$ is the amount of H^+^ millimoles in the initial HCl solution, $n_{\left(f,H^{+}\right)}$ is the amount of H^+^ millimoles in the final HCl solution after soaking the membrane, and *m*_d_ is the weight of dry membrane sample.

##### Water Uptake (WU%)

Water uptake (WU%) was determined by measuring the weight of wet and dry membranes, as reported by Nawn et al.^[^ ^8^ ^]^ The wet membrane was obtained, as above described, by soaking the material in double‐distilled water for 24 h, while the dry one by removing the adsorbed water under vacuum at 10^−2^ mbar for 48 h inside an argon dry box.

The water uptake was given by^[^ ^8^ ^]^

(6) $$\text{WU\%}=\;\frac{W_{\text{wet}}-W_{\text{dry}}}{W_{\text{dry}}}\times 100$$

where *W*_wet_ and *W*_dry_ are the weights of the wet and dry membranes, respectively.

##### Hydration Number (*λ*)

The hydration number (*λ*) is the average number of water molecules per quaternary ammonium polar group^[^ ^19^ ^]^ of membranes

(7) $$\lambda =\frac{1000\;\times \;(W_{\text{wet}}-\;W_{\text{dry}})}{IEC\;\times \;W_{\text{dry}}\;\times {\text{ MW}}_{H_{2}O}}$$

##### Chemical Stability

The chemical stability was determined by soaking the membrane in hydroxide form in a 1 m KOH solution at 60 °C for 336 h (14 days). The degradation degree was determined by measuring on time the ionic conductivity and ATR–FTIR spectra of membranes. The excess KOH solution was removed before each measurement by carefully washing the membrane with double‐distilled water at least three times.

## Conflict of Interest

The authors declare no conflict of interest.

## Supporting information

Supplementary Material

## Data Availability

The data that support the findings of this study are available on request from the corresponding author. The data are not publicly available due to privacy or ethical restrictions.
