# Supplementary material for: Interplay between Structure and Conduction Mechanism of Piperazinium‐Functionalized Poly[Ethylene Pyrrole/Ethylene Ketone/Propylene Ketone] Anion Conducting Membranes
Source: ChemSusChem. 2025 May 5;18(14):e202402765. doi: 10.1002/cssc.202402765 (PMC12270368; doi:10.1002/cssc.202402765)
Supplement: Supplementary file 1 — Supplementary Material [file CSSC-18-e202402765-s001.pdf]

SUPPORTING INFORMATION

Interplay between Structure and Conduction mechanism of Piperazinium Functionalized Poly[ethylene pyrrole/ethylene ketone/propylene ketone] Anion Conducting Membranes

Afaaf Rahat Alvi[a], Keti Vezzù[a,b], Francesco Lanero[a], Paolo Sgarbossa[a], Angeloclaudio Nale[a], Vito Di Noto[a,b]*

[a]Section of Chemistry for Technology, University of Padova, Department of Industrial Engineering, Via F. Marzolo 9, I-35131 Padova, Italy

[b]INSTM, Via F. Marzolo 9, I-35131 Padova, Italy

Experimental Section

Dynamic mechanical analysis

Dynamic mechanical analyses (DMA) are carried out with a TA Instruments DMA Q800 instrument, using the film/fiber tension clamp. The temperature spectra, in the range from room temperature to 200 °C at a rate of 3°C min^{-1}, are measured by subjecting a rectangular dry film sample (ca. 20 mm (height) x 6 mm (width) x 0.10 mm (thickness)) to an oscillatory sinusoidal tensile deformation at 1 Hz with an amplitude of 4 μm, and with a 0.02 N preload force. The sample is heated up using vaporized liquid nitrogen.

Results

S.1. Compositional Analysis

Table S1. IEC, water uptake (WU%), hydration number (λ) and conductivity at room temperature of membrane in I$^-$ and OH$^-$ form

$PKK_f(X)_g$	IEC$^{(a)}$ / meqg^{-1}	IEC$^{(tit)}$ /meqg^{-1}	WU%$^{(b)}$	Hydration Number (λ)$^{(c)}$	σ / mS·cm^{-1} at r.t.
P-FPKK$_{0.19}$(I)$_{0.18}$	1.19	/	12.66	5.92	0.06
P-FPKK$_{0.28}$(I)$_{0.27}$	1.54	/	11.21	5.00	0.01
P-FPKK$_{0.49}$(I)$_{0.45}$	2.02	2.10	27.29	7.50	0.21
P-FPKK$_{0.70}$(I)$_{0.66}$	2.44	/	26.41	6.30	0.11
P-FPKK$_{0.49}$(OH)$_{0.45}$	2.60	2.66	43.07	9.16	2.10

(a)IEC = ion exchange capacity determined by elemental analyses; IEC$^{(tit)}$ =ion exchange capacity obtained by titration experiments;

(b) WU % = $\frac{W_{wet}-W_{dry}}{W_{dry}}$ × 100 where W_{wet} and W_{dry} are the weights of the wet and dry membranes, respectively;

(c) λ = $\frac{1000 \times (W_{wet}-W_{dry})}{IEC \times W_{dry} \times M_{H_2O}}$ where M_{H_2O} is the molecular weight of water.

In order to determine whether single or multiple quaternization occurred, we determined the relative ratio between the number of moles of iodine (as iodide, determined by Mohr's titration and corresponding to the amount of quaternized nitrogens) and nitrogen (obtained from the elemental analysis of the polymer, see the table below for the data).

Elemental analysis data (extract of Table 1):

Sample	A/PK	%N	%C	%H	*%I*	*x*	*y+z*	*f*	*g*	*Q%*
P-FPKK$_{0.49}$(I)$_{0.45}$	1/1	8.58	52.6	6.03	25.9	0.49	0.61	0.49	0.45	91.8%

It is possible to determine the molar amount of each element per gram of polymer and the molar ration between iodine and nitrogen.

Calculated molar amount of the elements per gram and iodine/nitrogen molar ratio:

	C:H:N wt%	nN	nC	nH	nO	nI	nI/nN
**P-FPKK$_{0.49}$(I)$_{0.45}$**	52.6:6.03:8.58	0.612	4.38	5.98	0.429	0.204	0.333

Taking into account the presence of three nitrogen atoms in each pyrrolic repeat unit, a molar ratio of 0.333 is coherent with the presence of a single iodide anion per pyrrolic unit, thus confirming the mono-quaternization of each piperazine group.
To ascertain which is the preferential position for the methylation we conducted a 2D HSQC NMR experiment in the P-FPKK$_{0.49}$(I)$_{0.45}$ sample in DMF-d7 (the only deuterated solvent in which the polymer, even though sparingly, is soluble in this form). The spectrum (see below) shows the presence of both a spot at 2.19 ppm (**A** in the spectrum, $\delta^{13}C$ = 45.6 ppm) and at 3.44 ppm (**B** in the spectrum, $\delta^{13}C$ = 51.0 ppm) relative to methyl groups (blue spots) which are consistent with a 1,4-dimethyl-piperaziniumethyl species. In fact, the methyl groups of 4,4-dimethyl-1-phenyl-piperazinium iodide appear at 3.24 ppm (https://sdbs.db.aist.go.jp/CompoundView.aspx?sdbsno=7138, last accessed on 10/03/2025), while the methyl protons in 1,4-dimethyl-piperazine give a peak at 2.29 ppm (https://sdbs.db.aist.go.jp/CompoundView.aspx?sdbsno=2167, last accessed on 10/03/2025).
This rules out the quaternization in position 4, since it would lead to a 4,4-dimethyl-1-piperaziniumethyl group in which all the six protons of the CH_3 groups would be magnetically equivalent, giving rise to only one signal at around 3.2-3.5 ppm.
We can in fact hypothesize that upon reaction with iodomethane, both the nitrogens in 1 and 4 position can be methylated. Nevertheless, being the iodide ion a very weak base, when the nitrogen in 4 position is methylated, it retains the proton and is then protected against a second methylation reaction (being cationic and with no available lone pair). Only when the membrane is removed from iodomethane, washed and dried, the proton is released as HI giving the observed 1,4-dimethyl-piperaziniumethyl species.

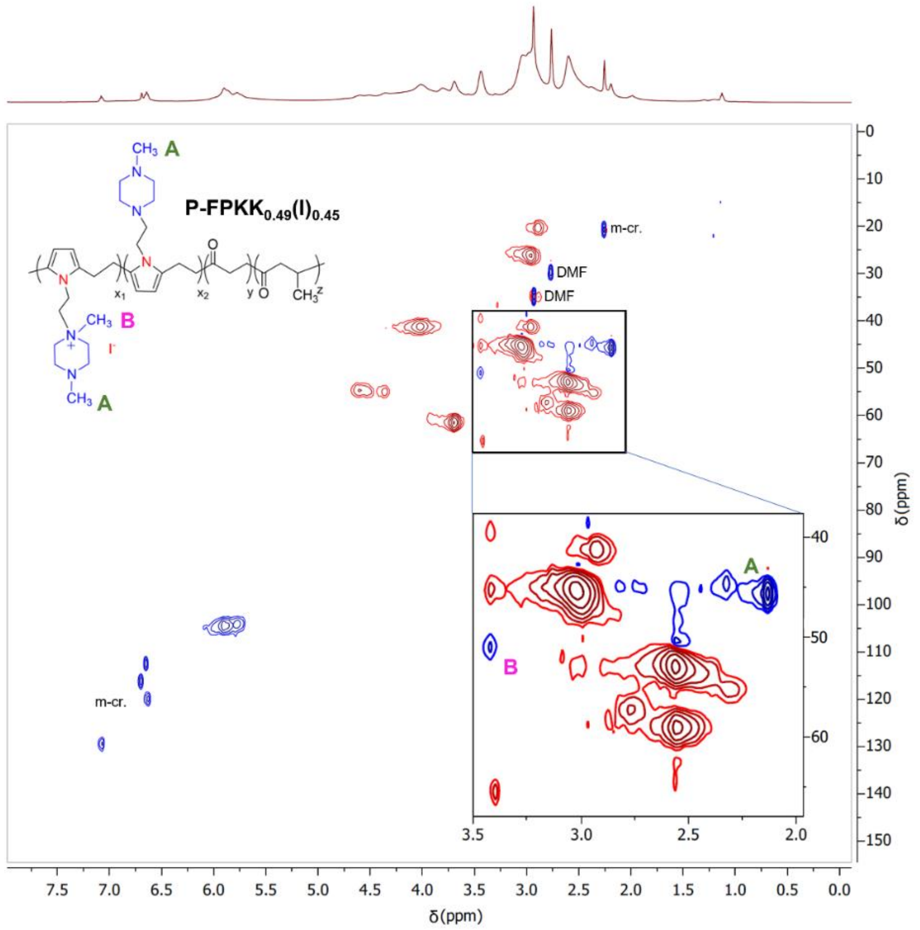

Figure S1. ^{1}H–^{13}C HSQC NMR spectra of P-FPKK$_{0.49}$(I)$_{0.45}$ in DMF-d7.

S.2. Thermal stability study: HR-TGA

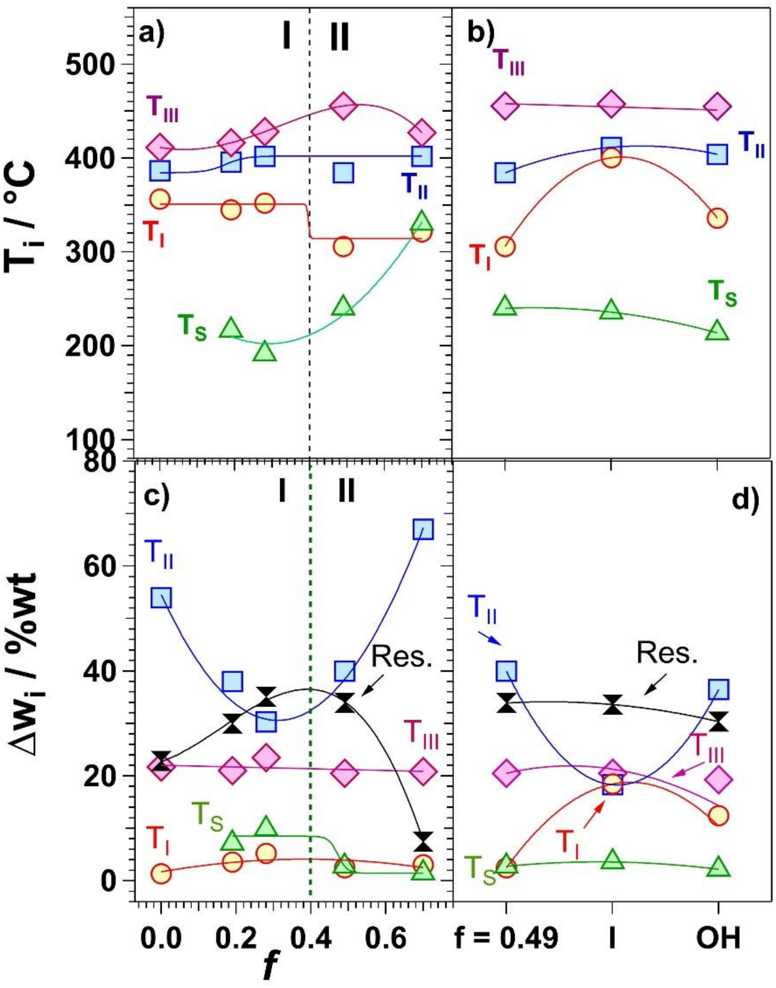

Figure S2. Dependence on *f* of degradation temperatures T_i (a), and mass elimination Δw_i (c) of P-FPKK$_f$. T_i (b) and Δw_i (d) of P-FPKK$_{0.49}$ and P-FPKK$_{0.49}$(X)$_{0.45}$ materials with X= I^-, OH^-

S.3. ATR-FTIR structural analysis

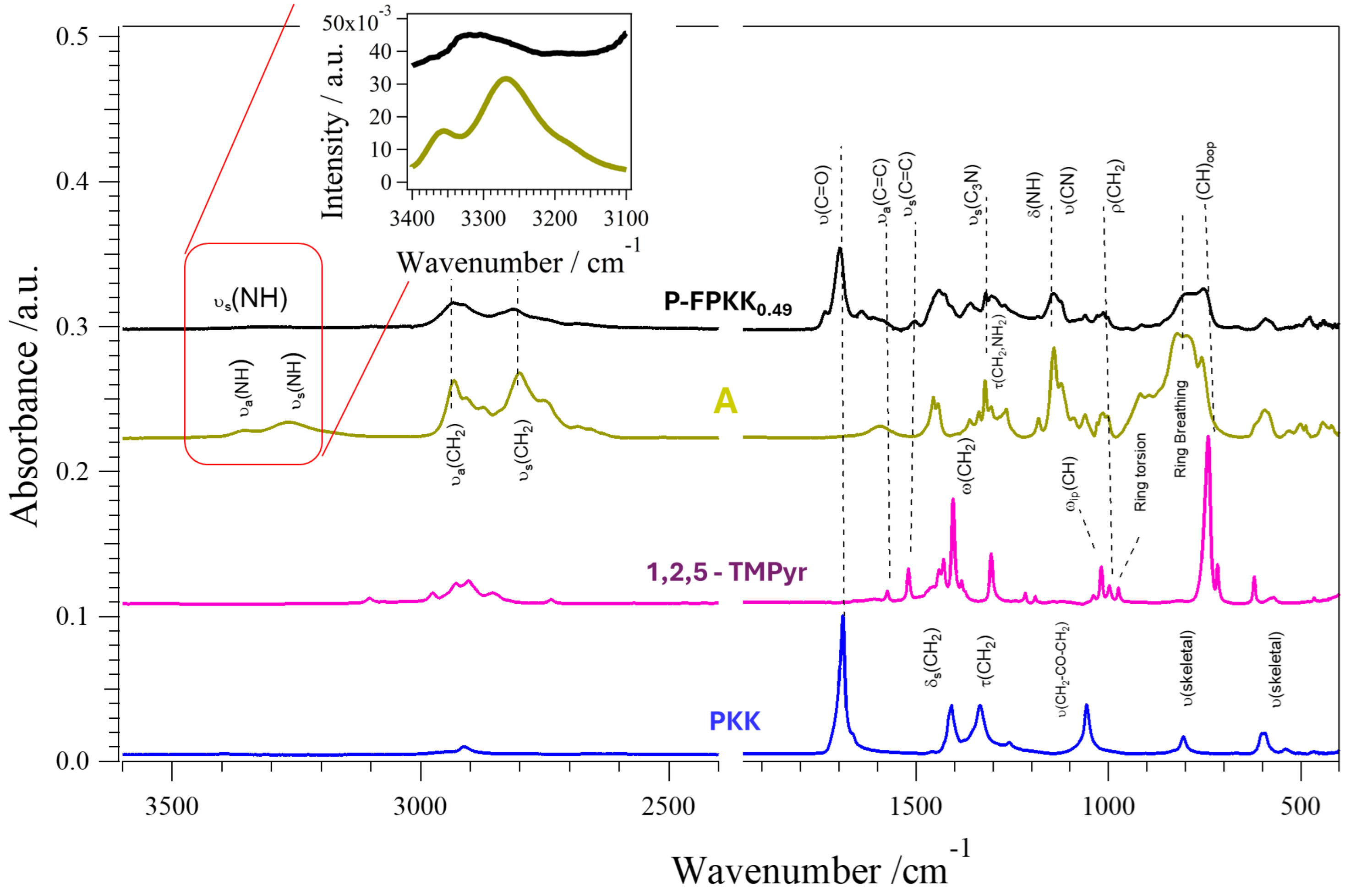

Figure S3. ATR-FTIR spectra of pristine polyketone (PKK-blue), 1,2,5-trimethylpyrrole (1,2,5-TMPyr-pink), 1-(2-aminoethyl)piperazine (A-green), and P-FPKK$_{0.49}$ (black) (the inset shows the ATR-FTIR profiles at $\nu > 3100$ cm^{-1})

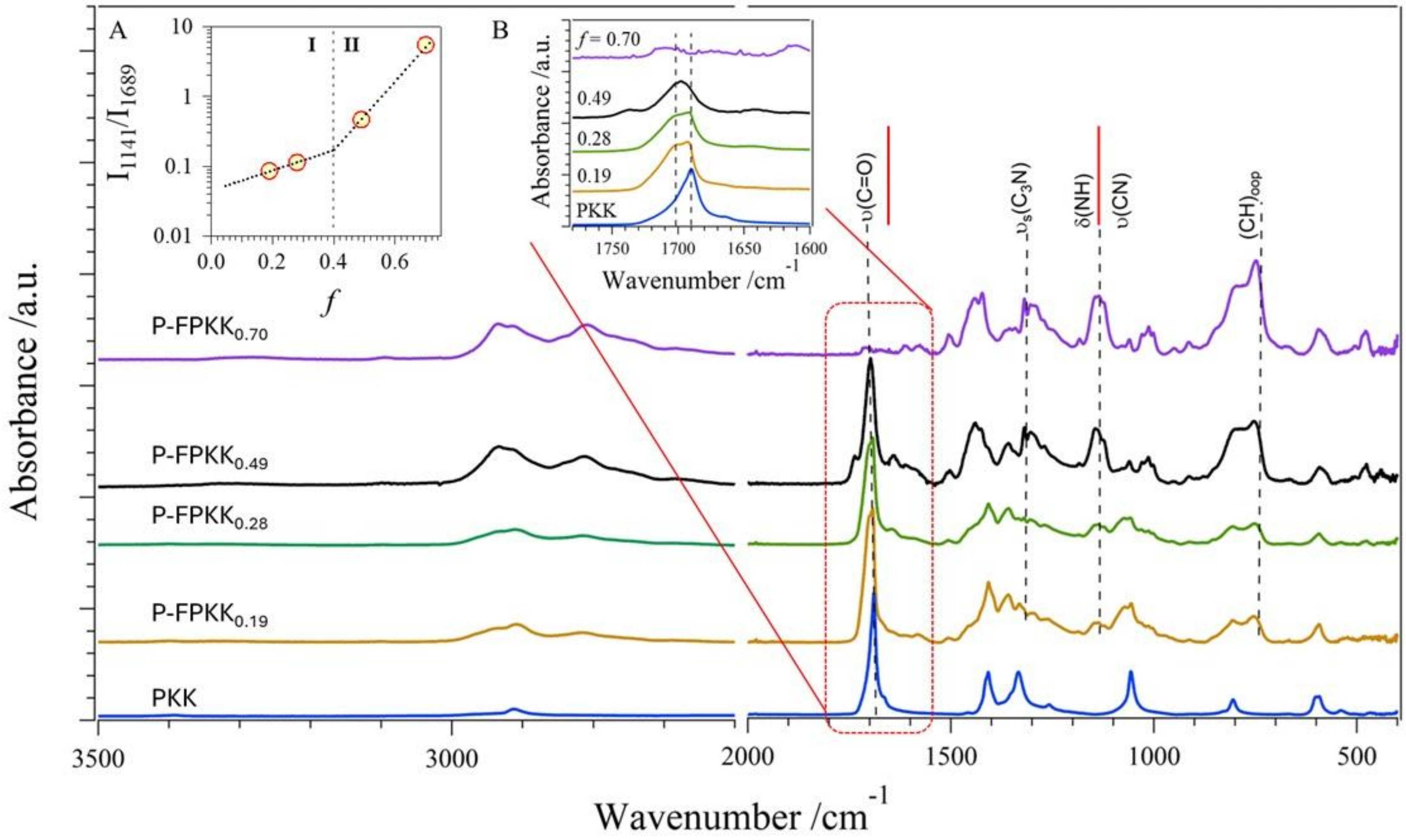

Figure S4. ATR-FTIR spectra of P-FPKK$_f$ on *f* (the inset shows the dependence on *f* of the ratio I_{1135}/I_{1689}, where I_{1135} and I_{1689} are the intensities of δ(NH) and ν(C=O) vibrational modes).

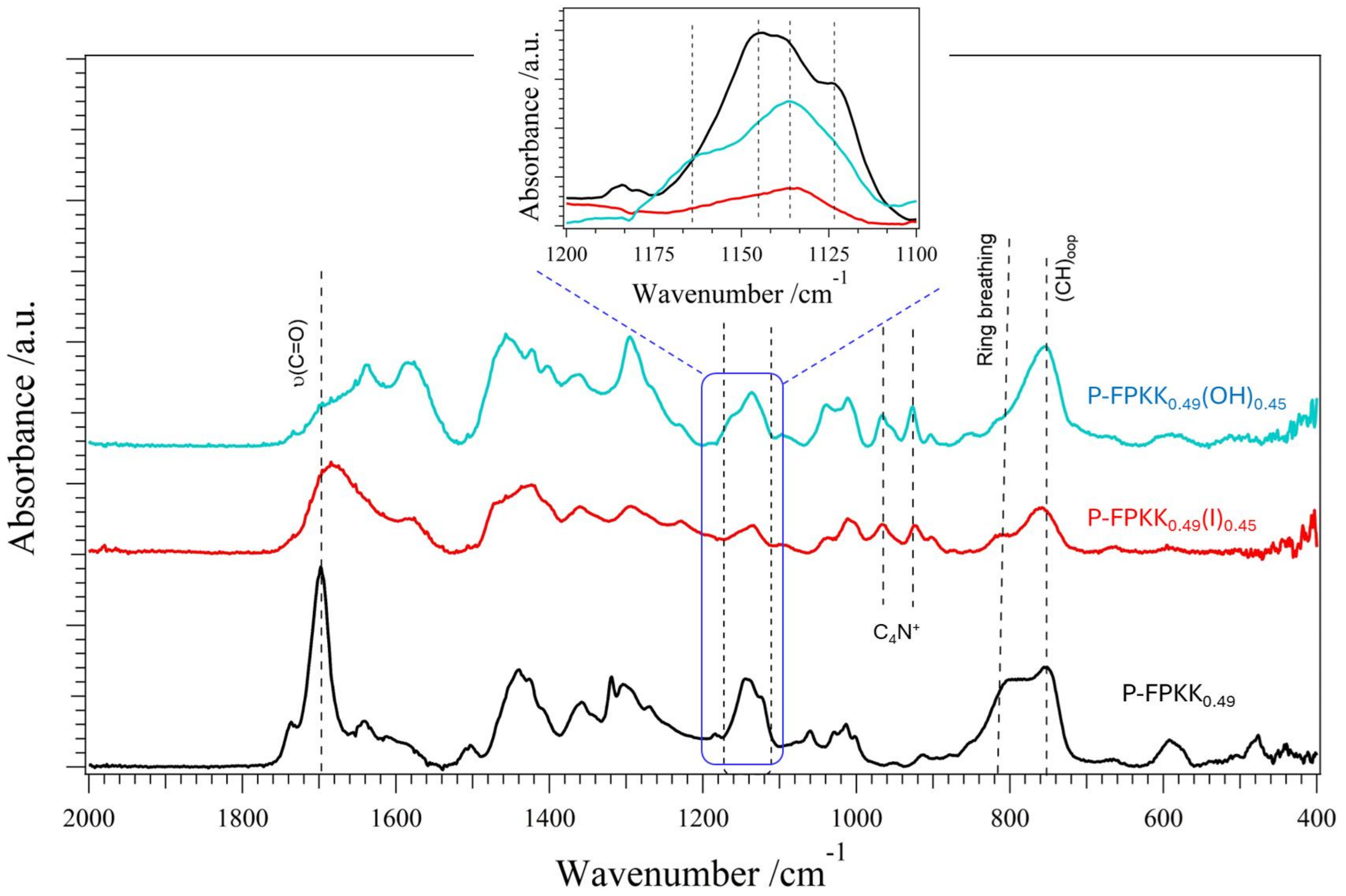

Figure S5. ATR-FTIR spectra of P-FPKK$_{0.49}$ and P-FPKK$_{0.49}$(X)$_{0.45}$ with X = I^-, OH^- (the inset shows the spectral region 1100-1200 cm^{-1}).

S.4. DMA analysis

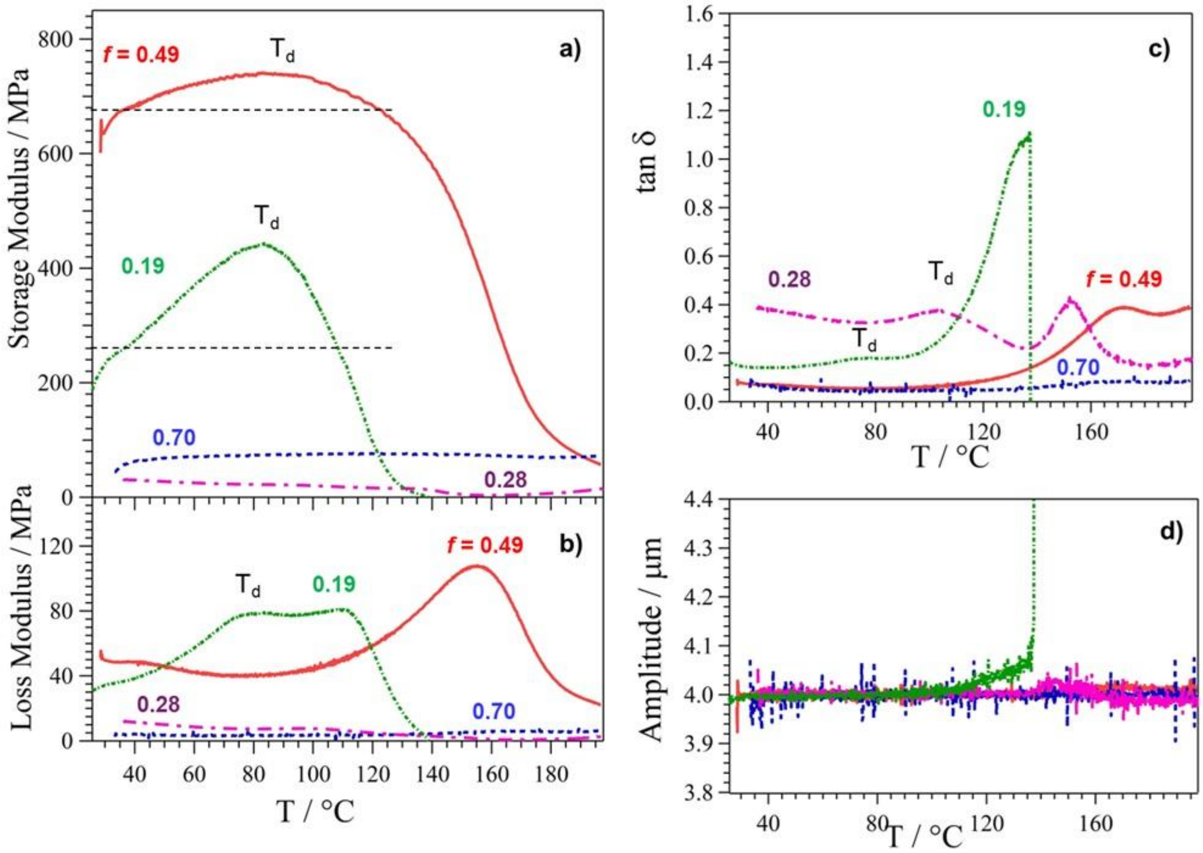

Figure S6. DMA studies for P-FPKK$_f$(I)$_g$ carried out from room temperature to 200°C with a temperature ramp of 3°C/min: a) storage modulus, b) loss modulus and c) tan δ at 1 Hz is shown. In d) the amplitude of the DMA measurements is reported. The black dashed horizontal line indicates the increase in storage modules.

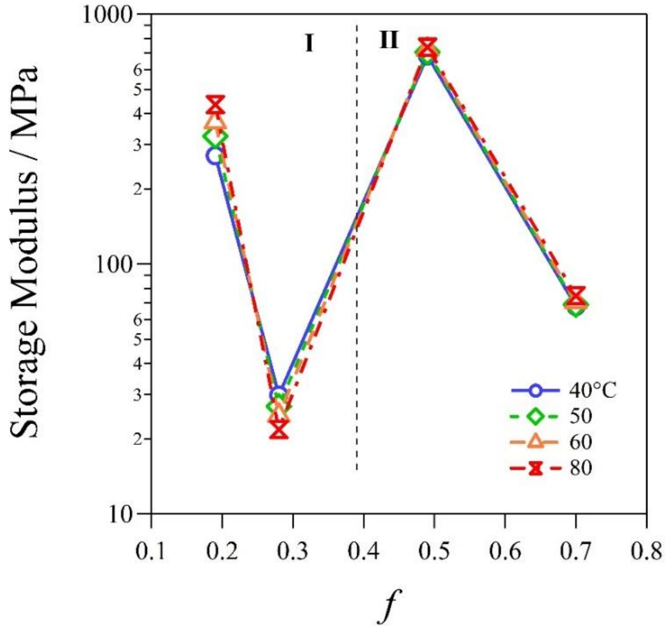

Figure S7. Storage Modulus as function of f and at different temperatures for P-FPKK$_f$(I)$_g$.

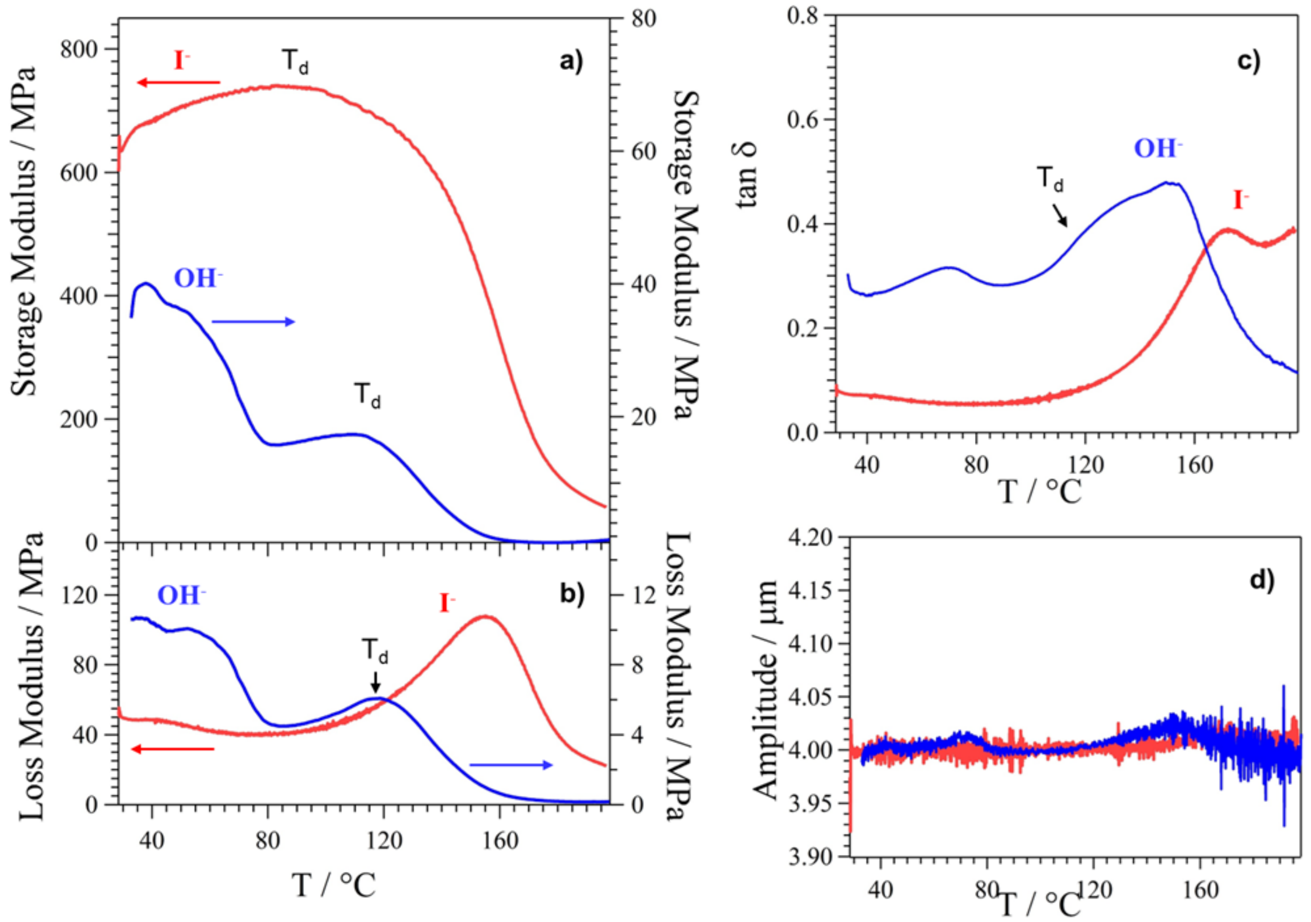

Figure S8. DMA studies for P-FPKK$_{0.49}$(X)$_{0.48}$ with X = I$^-$ and OH$^-$, carried out from room temperature to 200°C with a temperature ramp of 3°C/min: a) storage modulus, b) loss modulus and c) tan δ at 1 Hz is shown. In d) the amplitude of the DMA measurements is reported. The black dashed horizontal line indicates the increase in storage modules.

Figure S6 reports the DMA measurement results in terms of storage modulus, loss modulus, tan delta, and applied amplitude for the P-FPKK$_f$(I)$_g$ membranes tested starting from the wet condition. From Figure S6, an initial increase in the storage modulus can be observed, followed by step decreases.

The initial increase in storage modulus with temperature can be attributed to the reduction of water domains within the membranes due to heating under anhydrous gas flow. The decrease in water content promotes a conformational rearrangement of the polymer, leading to increased packing and, consequently, an increase in storage modulus. Concurrently, the decrease in storage modulus is strongly correlated with the thermal transitions of the material detected by MDSC, particularly the glass transition (T_g) and the melting of crystalline domains. Figure S7 shows the trend of the storage modulus as a function of frequency and temperature. It can be observed that, once again, the behaviour is similar to that observed for various other properties studied in these newly synthesized materials, such as water uptake, thermal transitions, and room-temperature conductivity. These results indicate a strong effect of functionalization, which influences crystallinity as well as inter- and intra-chain

dipole-dipole interactions due to the presence of side groups with dipole moments in opposite directions (T_d transition) [V. Di Noto, G. A. Giffin, K. Vezzù, G. Nawn, F. Bertasi, T.-h. Tsai, A. M. Maes, S. Seifert, E. B. Coughlin and A. M. Herring, Physical Chemistry Chemical Physics, 2015, 17, 31125-31139.]. Given the conductivity results of the hydroxylated membranes (Figure 7 in the main text), it was decided to study the DMA of the P-FPKK$_{0.49}$(OH)$_{0.48}$ membrane only. The comparison of the results for the hydroxylated membrane are reported in comparison with the iodinated one in Figure S8. As observed, the elastic modulus is lower in this case; however, the membrane remains mechanically stable up to 120°C, suggesting its potential application in AEMFC cells.
